# Improved quasi-invariance result for the periodic Benjamin–Ono–BBM equation

**DOI:** 10.1007/s00028-026-01237-3

**Published:** 2026-08-26

**Authors:** Justin Forlano

**Affiliations:** 1 School of Mathematics, Monash University, Melbourne, VIC 3800 Australia; 2 School of Mathematics, The University of Edinburgh, Edinburgh, UK; 3 The Maxwell Institute for the Mathematical Sciences, James Clerk Maxwell Building, Edinburgh, EH9 3FD UK

**Keywords:** Benjamin–Ono–BBM, Quasi-invariance, Gaussian measure, 35Q53, 76B15

## Abstract

We extend recent results of Genovese–Luca–Tzvetkov (2022) regarding the quasi-invariance of Gaussian measures under the flow of the periodic Benjamin–Ono–BBM (BO-BBM) equation to the full range where BO-BBM is globally well-posed. The main difficulty is due to the critical nature of the dispersion which we overcome by combining the approach of Coe–Tolomeo (2024) with an iteration argument due to Forlano–Tolomeo (2024) to obtain long-time higher integrability bounds on the transported density.

## Introduction

We consider the Benjamin–Ono–BBM (BO-BBM) equation:

1.1 $$\begin{aligned} {\left\{ \begin{array}{ll} \partial _tu +|D_x|\partial _tu+\partial _xu + \partial _x(u^2)=0 \\ u|_{t = 0} = u_0, \end{array}\right. } \qquad ( t, x) \in \mathbb {R}\times \mathbb {T}, \end{aligned}$$

where $|D_x|$ is the Fourier multiplier operator with multiplier |*n*|. The BO-BBM Eq. (1.1) arises as a model for the evolution of long-crested waves at the interface between two immiscible fluids. It is closely connected to the Benjamin–Ono (BO) equation [4, 38]:

1.2 $$\begin{aligned} \partial _tu +|D_x|\partial _xu+\partial _xu + \partial _x(u^2)=0, \end{aligned}$$

as, formally, (1.1) arises from replacing $\partial _x$ in the second term in (1.2) by $\partial _t$. This substitution has some physical significance related to the formal derivation of these models, see [5, 6].

To proceed with an analytical point of view, we may rewrite (1.1) as

1.3 $$\begin{aligned} \partial _tu =-(1+|D_x|)^{-1}\partial _x( u+ u^2). \end{aligned}$$

As compared to (1.2), the derivative in the nonlinearity of (1.1) has been completely ameliorated. In this sense, (1.1) is also referred to as the regularised Benjamin–Ono equation [1]. This moniker is partly merited by the following fact: whilst the well-posedness problem for the BO equation has historically been a difficult topic culminating in [21, 25] and the references therein, the well-posedness problem for BO-BBM (1.1) is much simpler.

To describe the well-posedness situation further, we need to set some notation. We define the Fourier transform by

$$\begin{aligned} \widehat{u} (n) =\frac{1}{2\pi } \int _{0}^{2\pi } e^{-\text {inx}} u(x) dx. \end{aligned}$$

and so that $\mathcal {F}\{ \partial _xu\}(n) = in \widehat{u}(n)$ and

$$\begin{aligned} \Vert u\Vert _{L^2(\mathbb {T})}^{2} = 2\pi \sum _{n\in \mathbb {Z}_{*}} |\widehat{u}(n)|^2. \end{aligned}$$

The mean $\int _{\mathbb {T}}udx$ is a conserved quantity for (1.1), and this allows us to, although not necessarily [2], restrict our discussions to the mean-zero Sobolev spaces $H^{\sigma }_{0}(\mathbb {T})$, with the norm

$$\begin{aligned} \Vert u\Vert _{H^{\sigma }}^{2} = \sum _{n\in \mathbb {Z}_{*}} |n|^{2\sigma }|\widehat{u}(n)|^2, \end{aligned}$$

where $\mathbb {Z}_{*}:= \mathbb {Z}\setminus \{0\}$. As $H^{\sigma }(\mathbb {T})$ is an algebra when σ>1/2 and the operator $(1+|D_x|)^{-1}\partial _x$ is bounded on $H^{\sigma }(\mathbb {T})$, we obtain local well-posedness for (1.3) in $H^{\sigma }(\mathbb {T})$. Global well-posedness for $\sigma \ge 1/2$ is also known, with the case σ>1/2 in [2, 29] following by combining the Brezis–Gallouët inequality with conservation of the energy

1.4 $$\begin{aligned} E(u) = \big (\Vert u\Vert _{L^2}^{2}+ 2\pi \Vert u\Vert _{H^{1/2}}^{2} \big )^{1/2}. \end{aligned}$$

This yields a super-exponential growth bound for the solutions:

1.5 $$\begin{aligned} \Vert u(t)\Vert _{H^{\sigma }} \lesssim \exp ( c( \log (1+\Vert u(0)\Vert _{H^{\sigma }})+ \Vert u(0)\Vert _{H^{1/2}}t)^2), \quad t\in \mathbb {R}. \end{aligned}$$

It turns out that in fact a mere exponential bound is available, which appears to be absent in the literature.

### Theorem 1.1

Let $\sigma >\frac{1}{2}$, $u_0 \in H^{\sigma }(\mathbb {T})$, and *u* be the unique global in time solution to (1.1) satisfying $u(0)=u_0$. Then, there exists $C_0,C_1>0$ such that for any $t\in \mathbb {R}$,

1.6 $$\begin{aligned} \Vert u(t)\Vert _{H^{\sigma }} \le C_0\Vert u_0\Vert _{H^{\sigma }} \exp ( C_1\Vert u_0\Vert _{H^{1/2}} |t|). \end{aligned}$$

We recall that in [19, Proposition 2.2], the authors showed that solutions to (1.1) satisfy^1^

1.7 $$\begin{aligned} \bigg | \frac{d}{dt} \Vert u(t)\Vert _{H^{s+\frac{1}{2}}}^{2} \bigg | \lesssim \Vert u(t)\Vert _{H^s}^2 \Vert \partial _xu (t)\Vert _{L^{\infty }} \end{aligned}$$

for any s≥0. By interpolation and the conservation of (1.4), (1.7) yields an exponential growth bound on solutions as in (1.6) when s>1, namely for $\sigma >\frac{3}{2}$. By refining the argument leading to (1.7), we are able to establish (1.6) for any $\sigma >\frac{1}{2}$.

The global well-posedness at the endpoint σ=1/2 was shown in [23] by a compactness argument from conservation of *E* and a Yudovich-type argument to recover uniqueness. Strictly speaking, [23] established this result on the real line $\mathbb {R}$ but the proof extends without additional complication to the periodic setting. We also point out that Theorem 1.1 also holds on $\mathbb {R}$. Thus, we have a well-defined global-in-time flow $u_0 \mapsto \Phi _{t}(u_0)$ on $H^{\sigma }(\mathbb {T})$ for any $\sigma \ge 1/2$. The well-posedness/ill-posedness of (1.3) is currently open for σ<1/2. See [2] for a mild form of ill-posedness (failure of $C^2$-regularity for the solution map) when σ<0.

Our interest in (1.1) is to improve upon the known regularity ranges under which Gaussian measures remain absolutely continuous under the flow of (1.1) and to the point at which agrees with the well-posedness theory. In the setting of Hamiltonian PDEs, this direction was initiated by Tzvetkov [45] and has since seen a remarkable amount of progress [10, 12, 13, 15–20, 22, 27, 32–36, 39–41, 44, 45]. In particular, these results together show that there is a delicate balance between positive results in this area (quasi-invariance) and the amount of dispersion and/or structure in the given equation. In this vein, BO-BBM (1.1) is an interesting model for this study as the amount of dispersion seems to be borderline for positive quasi-invariance results to hold; see [20], Theorem 1.2, and the ensuing discussion below.

Let us first describe this quasi-invariance problem in more detail, focusing on the situation for the mean-zero flow of BO-BBM (1.1). Given $s\in \mathbb {R}$, we consider the Gaussian measures $\mu _s$ formally prescribed as

$$\begin{aligned} d\mu _s = Z_{s}^{-1} e^{-\frac{1}{2}\Vert u\Vert _{H^{s+1/2}}^{2}} du, \end{aligned}$$

and rigorously defined as the law of the random Fourier series

1.8 $$\begin{aligned} u_{0}^{\omega } (x) = \sum _{n\in \mathbb {Z}_{*}} \frac{g_n (\omega )}{|n|^{s+1/2}} e^{inx}, \end{aligned}$$

where $\{g_{n}(\omega )\}_{n\in \mathbb {Z}_{*}}$ are a family of complex-valued standard normal random variables on some probability space $(\Omega , \mathcal {F}, \mathbb {P})$ and conditioned so that $g_{-n}(\omega ) = \overline{g_n(\omega )}$. These Gaussian measures are supported on the Sobolev spaces $H^{\sigma }_{0}(\mathbb {T})$ for any σ<s. Previously, Genovese–Luca–Tzvetkov [20] have shown that for every s>1,

1.9 $$\begin{aligned} (\Phi _{t})_{\#}\mu _{s} \sim \mu _s \quad \text {for all} \quad t\in \mathbb {R}, \end{aligned}$$

where ∼ denotes the mutual absolute continuity of measures, namely $(\Phi _{t})_{\#}\mu _{s} \ll \mu _s$ and $\mu _s \ll (\Phi _{t})_{\#}\mu _{s}$. The property (1.9) is called quasi-invariance.

The authors in [20] were able to say more than (1.9), as we describe now. Given R>0, we denote by $B_{R}$ the closed ball centred at zero of radius *R* in the energy space; namely,

1.10 $$\begin{aligned} B_{R} : =\big \{ u\in H_0^{1/2}(\mathbb {T})\,:\, E(u)\le R\big \}, \end{aligned}$$

and define the localised measure

1.11 $$\begin{aligned} d\mu _{s,R} = \textbf{1}_{B_R}(u) d\mu _s. \end{aligned}$$

The addition of the (invariant) cut-off $\textbf{1}_{B_R}$ allowed them to show that the transported density (Radon–Nikodym derivative)

1.12 $$\begin{aligned} f_{t,R} : = \frac{d(\Phi _{t})_{*}\mu _{s,R}}{d\mu _{s,R}} \end{aligned}$$

enjoys higher $L^p$-integrability, than merely belonging to $L^{1}(d\mu _{s,R})$. However, as a consequence of the critical nature of the dispersion in (1.3), the integrability index p=p(t,R) depends on t>0 and has the property that p(|t|,R)→1 as $|t|\rightarrow +\infty$. Interestingly, this deterioration of the exponent does not occur for models with even slightly stronger dispersion, such as for the fractional BBM equations

1.13 $$\begin{aligned} \partial _tu +|D_x|^{\beta }\partial _tu+\partial _xu + \partial _x(u^2)=0 \end{aligned}$$

with β>1 (where β=1 corresponds to (1.1)); see [19]. It is in this sense that we see BO-BBM (1.1) as a model with a critical amount of dispersion for quasi-invariance to hold. Note that a critical aspect also appears for nonlinear wave equations [22, 36].

Our goal is to extend the results of [20] to the full range s>1/2 where BO-BBM (1.1) is well-posed.

### Theorem 1.2

Given $s>\frac{1}{2}$ and $t\in \mathbb {R}$, the transported measure $(\Phi _{t})_{*}\mu _{s}$ is mutually absolutely continuous with respect to the Gaussian measure $\mu _s$. Moreover, for each t>0 and R>0 there exists p=p(|t|,R)>1 such that p(|t|,R)→1 as $|t|\rightarrow \infty$ and constants $C_1(R), C_2(R)>0$, for which the Radon–Nikodym derivative $f_{t,R}$ defined in (1.12) satisfies

1.14 $$\begin{aligned} \Vert f_{t,R}\Vert _{L^{p}(d\mu _{s,R})} \le C_1(R)e^{C_2(R)t} . \end{aligned}$$

The regularity restriction of s>1 in [20] arises from an energy estimate on $\log f_{t,R}$, which is essentially (1.7). More precisely, it is enough to establish the $L^p(\mu _s)$ boundedness for the right-hand side of (1.7) at time t=0. This reduction allows to use the probabilistic properties of the initial data. Here, in order to overcome the logarithmic divergence from the $H^{s}-$factors, one uses the $\mu _s$ almost sure finiteness of $\Vert \partial _xu\Vert _{L^{\infty }}$ which imposes s>1.

Our strategy to go beyond the analysis in [20] is to adapt the recent approach of [12] which allows to use probability in a more complete way as well as make use of a symmetrisation argument in (3.6). More precisely, we can exploit *multilinear* probabilistic oscillations by controlling the left-hand side of (1.7), rather than passing to the right-hand side which only allows to use probability at a *linear* level. The main difficulty, which is also the key to technical result leading to Theorem 1.2, is then to establish the exponential integrability of $\log f_{t,R}$ (Lemma 3.3), and the variational formula [3, 7, 46] provides a sharp way to prove this.

Once we have in hand Lemma 3.3, it is possible to obtain the quasi-invariance using an argument from [36], which is based on tracking the growth of the measure of a transported set, and from this obtain the higher integrability of the density (1.14) using [10, Proposition 3.5]. We instead give an alternative, but equivalent, analysis focusing purely on the density. However, due to the critical dispersion of BO-BBM (1.1) it is not enough to simply apply the method in [12]. Indeed, we are not able to obtain (3.11) as directly as one would in [12] because of the short-time restriction in Lemma 3.4. We need to go one step beyond this by carefully iterating these bounds on short-time intervals to reach long times (Proposition 3.5). For this step, we adapt an argument from [17]. At a high level, we advocate that this strategy arising from combining ideas from [12, 17] provides an effective way to analyse the low-regularity quasi-invariance problem for equations with a borderline amount of dispersion.

As discussed in [20], a complete solution to the programme initiated in [45] would constitute finding a formula for the transported density with respect to the unrestricted Gaussian measure $\mu _s$. This has only been achieved in a few works [15, 19, 27, 40] and relies on more dispersion, namely on good energy estimates for solutions *with* smoothing. It seems to be an interesting but difficult problem to determine if (i) one could remove the localisation $B_{R}$ in (1.14) and (ii) obtain a formula for $f_{t,R}$, and the density with respect to $\mu _s$. In particular, we can no longer reduce to time t=0 and thus it is not clear how to guarantee the finiteness of the right-hand side of (1.7). Indeed, there is no multilinear smoothing in the nonlinear term in (1.3), even under randomisation: if *u* were replaced by $u_0^{\omega }$ given in (1.8). The situation becomes even more difficult for the regularity range $\frac{1}{2}<s\le 1$ considered here since we need to consider $\frac{d}{dt} \Vert u(t)\Vert _{H^{s+\frac{1}{2}}}^2$ itself and cannot reduce to using (1.7).

The analysis in this paper simplifies dramatically if one is only interested in the qualitative statement of quasi-invariance as in (1.9) and not the higher integrability property of the transported density. Indeed, in this case it is possible to instead argue solely as in [12] by replacing the cut-off in (1.11) by a cut-off to a ball in $H^{\sigma }_{0}(\mathbb {T})$ for $\frac{1}{2}<\sigma <s$. Namely, there is no need to use an argument as in Proposition 3.5 as there is no longer an upper bound restriction on the parameter λ in Lemma 3.3. However, such an approach does not say anything about higher integrability of the density and thus is not in the spirit of identifying a formula for the density.

The aforementioned lack of nonlinear smoothing under randomisation also complicates efforts to extend Theorem 1.2 to some range s≤1/2. The first step here would be to construct local in time solutions almost surely with respect to $\mu _s$, but a first-order (Da–Prato Debussche) expansion of the solution does not work due to the presence of high-low interactions. Thus, even almost sure *local* well-posedness on the support of the Gaussian measure $\mu _s$ is non-trivial. In the presence of an almost surely globally well-defined flow with respect to $\mu _s$, we conjecture that quasi-invariance fails when $0<s\le \frac{1}{2}$ and that the transported measure should be singular with respect to $\mu _s$. To give some evidence for this, we recall that Coe–Tolomeo [12] provided a benchmark test as to when quasi-invariance may or may not hold. Their criterion, in our setting, amounts to computing the quantity

$$\begin{aligned} \text {QI}_{s,N}(t):=\mathbb {E}\bigg [ \bigg | \int _{0}^{t} Q_{s,N}( S(-t') u_0^{\omega })dt'\bigg |^{2}\bigg ], \end{aligned}$$

where $Q_{s,N}$ is defined in (3.3) and $S(t):=e^{t(1+|D_x|)^{-1}\partial _x}u_0^{\omega }$ is the linear solution for (1.3) with the Gaussian random initial data (1.8). If $\text {QI}_{s,N}$ is finite uniformly in *N*, we may expect quasi-invariance, whilst if it is not, we may expect singularity. In Sect. 4.3, we prove the following:

### Proposition 1.3

Let $0< s \le \frac{1}{2}$. For all *N* sufficiently large and |t|≲1, it holds that

1.15 $$\begin{aligned} QI _{s,N}(t) \gtrsim {\left\{ \begin{array}{ll} t^{2} N^{1-2s} \quad &  \text {if} \quad s<\frac{1}{2},\\ t ^{2}(\log N)^2 \quad &  \text {if} \quad s=\frac{1}{2}. \end{array}\right. } \end{aligned}$$

Note that we omitted the case s=0 in Proposition 1.3 as formally we expect the measure $Z^{-1} e^{-E(u)^2}du$, where *E* is as in (1.4), to be invariant under the flow of (1.3). Of course, it is unclear if there is any well-defined dynamics almost surely on the support of this measure.

## Preliminaries

For $x\in \mathbb {R}$, we define $\langle x \rangle = \sqrt{1+x^2}$. We let $\mathbb {N}=\{1,2,\ldots \}$ and $\mathbb {N}_0 =\mathbb {N}\cup \{0\}$. Given $n_1,n_2,n_3\in \mathbb {Z}$, we let $n_{123}:=n_1+ n_2+n_3$, and, for j=1,2,3, denote by $n_{(j)}$ the *j*th largest of these numbers in magnitude so that $|n_{(1)}|\ge |n_{(2)}|\ge |n_{(3)}|$. Given $1\le p\le \infty$, we let $1\le p'\le \infty$ denote its Hölder conjugate satisfying $\frac{1}{p}+\frac{1}{p'}=1$.

We define Littlewood–Paley operators as follows: Let $\eta \in C^{\infty }_{c}(\mathbb {R})$ be a smooth bump function such that $0\le \eta \le 1$, $\eta (\xi )=1$ for $|\xi |\le 1$ and $\eta (\xi )=0$ for $|\xi |\ge 2$. We define $\phi (\xi ):=\eta (\xi )-\eta (2\xi )$, and for $N\in 2^{\mathbb {Z}}$, we set $\phi _{N}(\xi )=\phi (\xi /N)$. For $N\in 2^{\mathbb {Z}}$, we define projectors

$$\begin{aligned} \widehat{\textbf{P}_N f}(n) = \phi _{N}(n)\widehat{f}(n), \quad \widehat{\textbf{P}_{\le N} f}(n) = \eta (\tfrac{n}{N})\widehat{f}(n), \quad \widehat{ \textbf{P}_{>N} f}(n)=(1-\eta (\tfrac{n}{N})) \widehat{f}(n). \end{aligned}$$

We define the wider projector $\widetilde{\textbf{P}}_{N}:= \textbf{P}_{\le 8N}-\textbf{P}_{\le N/8}$, which has the property that $\widetilde{\textbf{P}}_{N} \textbf{P}_{N} = \textbf{P}_{N}$. We also use the notation $u_{\ll N}:= \textbf{P}_{\le 2^{-10}N}u$ and $u_{\gtrsim N}:= u-u_{\ll N}$.

Given $N\in \mathbb {N}$, we define the projected Gaussian measures $\mu _{s,N}:= (\textbf{P}_{\le N})_{\#}\mu _s$ which are Gaussian measures in $\mathbb {R}^{2N}$, and we define $\mu _{s,N}^{\perp }$ as the law of the random Fourier series

$$\begin{aligned} u_{N}^{\perp } (x;\omega ) = \sum _{|n|>N} \frac{g_{n}(\omega )}{ |n|^{s+1/2}}e^{inx}. \end{aligned}$$

### Lemma 2.1

Let $s>\frac{1}{2}$ and $N\in \mathbb {N}$. Then, for any M≥1, it holds that

2.1 $$\begin{aligned} \mu _{s,N}^{\perp } \big ( \Vert u_{N}^{\perp }\Vert _{H^{1/2}_0(\mathbb {T})}^2 >M\big ) \lesssim M^{-2}N^{-2s+1} \end{aligned}$$

where the implicit constant is uniform in *M* and *N*.

### Proof

We have

$$\begin{aligned} \mathbb {E}[\Vert u_{N}^{\perp }\Vert _{H^{1/2}_0(\mathbb {T})}^{2} ]&\lesssim \mathbb {E}\bigg [ \sum _{|n|>N} \frac{|g_n|^2}{|n|^{2s}}\bigg ] \lesssim \sum _{|n|>N} \frac{1}{|n|^{2s}} \lesssim N^{-2s+1}, \end{aligned}$$

at which point, (2.1) follows from Markov’s inequality. □

We can obtain an exponential decay rate in (2.1) but the polynomial one here will be sufficient.

## Localised density bounds

Given $N\in \mathbb {N}$, we define the truncated version of (1.3):

3.1 $$\begin{aligned} \partial _tu_{N} = -(1+|D_x|)^{-1}\partial _x\textbf{P}_{\le N} ( u_N + u_N^2), \quad u_{N}|_{t=0} = \textbf{P}_{\le N} u_0 \end{aligned}$$

Similar to (1.1), for every fixed $N\in \mathbb {N}$, (3.1) is globally well-posed in $H^{\sigma }$, for $\sigma >\frac{1}{2}$, with bounds on the Sobolev norms of the solutions that are uniform in *N*. This can be proved by repeating the arguments in [29] and using the $L^2$-boundedness of $\textbf{P}_{\le N}$. We denote the flow of (3.1) by $\Phi ^{N}_{t}$. Note that if $u_N$ solves (3.1), then $u_N\in C^{\infty }(\mathbb {R}\times \mathbb {T})$, which justifies the ensuing computations.

As the truncated Eq. (3.1) is a finite-dimensional Hamiltonian system (in the Fourier coefficients for $u_N$), Liouville’s theorem implies that the Lebesgue measure $L_N$ on $\mathbb {R}^{2N}$ is invariant under $\Phi _{t}^{N}$. Thus, for every $t\in \mathbb {R}$,

3.2 $$\begin{aligned} d(\Phi ^{N}_{t})_{\#} \mu _{s,N}&= f_{t}^{N}(u) d\mu _{s,N} := \exp \Big ( - \tfrac{1}{2}\Vert \Phi ^{N}_{t}(u)\Vert _{H^{s+1/2}}^{2} + \tfrac{1}{2} \Vert u\Vert _{H^{s+1/2}}^{2} \Big )d\mu _{s,N}, \end{aligned}$$

where $f_{t}^{N}$ is the transported density. By the fundamental theorem of calculus, we have

$$\begin{aligned} -\tfrac{1}{2}\Vert \Phi ^{N}_{t}(u)\Vert _{H^{s+1/2}}^{2} + \tfrac{1}{2} \Vert u\Vert _{H^{s+1/2}}^{2} = \int _{0}^{-t} \mathcal {Q}_{s,N}(\Phi _{t'}^{N}(u))dt', \end{aligned}$$

where $\mathcal {Q}_{s,N}(u): =\mathcal {Q}_{s,N}(u,u,u)$ is the trilinear operator

3.3 $$\begin{aligned} \mathcal {Q}_{s,N}(u)&: = - \frac{d}{dt} \bigg (\frac{1}{2} \Vert \Phi _{t}^{N}(u)\Vert _{H^{s+1/2}}^2 \bigg ) \Bigg \vert _{t=0}. \end{aligned}$$

From (3.1), the identity $(1+|D_x|)^{-1} |D_x| = 1-(1+|D_x|)^{-1}$ and for $u_{N}:=\textbf{P}_{\le N} u$, we then have

3.4 $$\begin{aligned} \begin{aligned} \mathcal {Q}_{s,N}(u)&= \langle |D_x|^{2s+1} u_{N}, (1+|D_x|)^{-1} \partial _xu_{N} \rangle + \langle |D_x|^{2s+1} u_N, (1+|D_x|)^{-1} \partial _x(u_N^2) \rangle \\&= \langle |D_x|^{2s} u_N, \partial _x(u_N^2) \rangle - \langle |D_x|^{2s} u_N, (1+|D_x|)^{-1} \partial _x(u_N^2) \rangle . \end{aligned} \end{aligned}$$

Notice that the contribution from the linear part in (3.4) vanishes identically since

$$\begin{aligned}&\langle |D_x|^{2s+1} u_{N}, (1+|D_x|)^{-1} \partial _xu_{N} \rangle \\&= \langle |D_x|^{s+\frac{1}{2}}(1+|D_x|)^{-\frac{1}{2}}u_N, \partial _x|D_x|^{s+\frac{1}{2}}(1+|D_x|)^{-\frac{1}{2}}u_N \rangle =0. \end{aligned}$$

By a symmetrisation argument, we write (3.4) this more favourably as

3.5 $$\begin{aligned} \mathcal {Q}_{s,N}(u) = \mathcal {Q}^{(1)}_{s,N}(u) + \mathcal {Q}^{(2)}_{s,N}(u), \end{aligned}$$

where we define the trilinear operators $\mathcal {Q}^{(j)}_{s,N}(u):= \mathcal {Q}^{(j)}_{s,N}(u,u,u)$, j=1,2, for $u\in \Pi _{\le N}L^2$, by

3.6 $$\begin{aligned} \mathcal {Q}^{(1)}_{s,N}(u_1,u_2,u_3)&= \sum _{\begin{array}{c} n_1 +n_2+n_3=0 \\ 0<|n_j|\le N, j=1,2,3 \end{array} } i\Psi _{s}(\overline{n}) \widehat{u}_1 (n_1) \widehat{u}_2(n_2) \widehat{u}_3(n_3) \end{aligned}$$

3.7 $$\begin{aligned} \mathcal {Q}^{(2)}_{s,N}(u_1,u_2,u_3)&= \langle |D_x|^{2s}u_1, (1+|D_x|)^{-1}\partial _x(u_2 u_3) \rangle \end{aligned}$$

and

3.8 $$\begin{aligned} \Psi _{s}(\overline{n}):= \Psi _{s}(n_1,n_2,n_3): = |n_1|^{2s+1}{{\,\textrm{sgn}\,}}(n_1) + |n_2|^{2s+1}{{\,\textrm{sgn}\,}}(n_2)+|n_3|^{2s+1}{{\,\textrm{sgn}\,}}(n_3), \end{aligned}$$

The multilinear operator $\mathcal {Q}^{(1)}_{s,N}$ is worse behaved as compared to $\mathcal {Q}^{(2)}_{s,N}$ because it lacks the factor $(1+|D_x|)^{-1}$. Nonetheless, the symmetrisation in $\mathcal {Q}^{(1)}_{s,N}$ allows us to always place the derivative $\partial _x$ onto the lowest frequency term, in the following sense.

### Lemma 3.1

Let $s>\frac{1}{2}$ and $n_1,n_2,n_3\in \mathbb {Z}_{*}$ such that $n_1 +n_2+n_3=0$. Then, either (i) $|n_1|\sim |n_2|\sim |n_3|$ or (ii) $|n_{(2)}| \gg |n_{(3)}|$ and hence $|\Psi _{s}(\overline{n})| \lesssim |n_{(1)}|^{2s} |n_{(3)}|.$

### Proof

By symmetry, we may assume that $|n_1|\ge |n_2|\ge |n_3|$. Thus, when $|n_2|\gg |n_3|$, we must have ${{\,\textrm{sgn}\,}}(n_1)=-{{\,\textrm{sgn}\,}}(n_2)$, so by the mean value theorem,

$$ |\Psi _{s}(\overline{n})| \le ||n_1|^{2\,s+1} - |(-n_2)|^{2\,s+1}| +|n_3|^{2\,s+1} \lesssim |n_1|^{2\,s}|n_3| + |n_3|^{2\,s+1}\sim |n_1|^{2\,s}|n_3|. $$

□

We note that since *E* in (1.4) is a conserved quantity on the support of $\mu _s$, $B_{R}$ is an invariant set under $\Phi ^{N}_{t}$; namely, $(\Phi ^{N}_{t})^{-1}(B_R)=B_{R}$ for any $t\in \mathbb {R}$.

### Remark 3.2

We note that the density $f_{t}^N$ localised to $B_{R}$ always has finite $L^p(d\mu _s)$-moments, albeit with a severe loss in uniformity in *N*. Indeed, for $u\in B_R$, we have $\Phi _{t}^{N}(u)\in B_{R}$ and thus Sobolev embeddings imply

$$\begin{aligned} |\mathcal {Q}_{s,N}(\Phi _{t}^{N}(u))| \lesssim N^{2s+\frac{1}{2}} \Vert \Phi _{t}^{N}(u)\Vert _{H^{\frac{1}{2}}}^{3} \lesssim N^{2s+\frac{1}{2}}R^{\frac{3}{2}}. \end{aligned}$$

Our goal is to obtain bounds which are uniform in *N*.

The main ingredient in this article is the following exponential integrability statement.

### Lemma 3.3

Let $\frac{1}{2} <s\le 1$, $N\in \mathbb {N}$, and R>0. Then, there exist constants $c_0>0$ and A>1 independent of both *R* and *N* such that for all $0\le \lambda \le c_0 R^{-\frac{1}{2}}$, it holds that

3.9 $$\begin{aligned} \sup _{N\in \mathbb {N}} \int \exp \big ( \lambda |\mathcal {Q}_{s,N}(u)| \textbf{1}_{B_{R}}(u)\big ) d\mu _{s,N} \le e^{C(R) \max (\lambda , \lambda ^{A})}. \end{aligned}$$

We postpone the proof of Lemma 3.3 to Sect. 4. Using Lemma 3.3, we obtain the following uniform in *N*, short-time $L^p$ bounds on the densities.

### Lemma 3.4

Let $\frac{1}{2} <s\le 1$, $N\in \mathbb {N}$, and R>0. Then, there exists $c_0>0$ independent of both *R* and *N* such that for any $t\in \mathbb {R}$ and $1\le p<\infty$ satisfying $0\le p| t|\le c_0 R^{-\frac{1}{2}}$, there exists C(R,p)>0 such that

3.10 $$\begin{aligned} \sup _{N\in \mathbb {N}} \Vert f_{t}^{N}\textbf{1}_{B_{R}}\Vert _{L^p(d\mu _{s,N})} \le e^{C(R) \max ( |t|p , (|t|p)^A)}. \end{aligned}$$

### Proof

The proof largely follows that of [12, Proposition 3.6]; however, as $B_{R}$ is invariant under $\Phi ^{N}_{t}$, there are some simplifications. For the convenience of the reader, we provide the details. First, we reduce the proof of the bound (3.10) for $f_{t}^N \textbf{1}_{B_R}$ in such a way that it remains to apply Lemma 3.3. Let $I_{t}=[0,t]$ for t≥0 and $I_t= [t,0]$ for t<0. Let t≠0. By the formula (3.2), the group property of the flow $\Phi ^{N}_t$, Jensen’s inequality, Fubini’s theorem, and the invariance of $B_{R}$, we have

$$\begin{aligned} \Vert f_{t}^{N} \textbf{1}_{B_R}\Vert _{L^p (\mu _{s,N})}^{p}&= \int (f_{t}^{N}(u))^{p-1} \textbf{1}_{B_{R}}(u) f_{t}^{N}(u) d\mu _{s,N} \\&= \int (f_{t}^{N}(\Phi _{t}^{N}(u)))^{p-1} \textbf{1}_{B_{R}}(u) d\mu _{s,N}(u) \\&= \int \exp \bigg ( -(p-1)\int _{0}^{-t} \mathcal {Q}_{s,N}( \Phi _{t+\tau }^{N}(u))d\tau \bigg ) \textbf{1}_{B_{R}}(u)d\mu _{s,N}(u)\\&= \int \exp \bigg ( \frac{t}{|t|}(p-1)\int _{I_t}\mathcal {Q}_{s,N}( \Phi _{\tau }^{N}(u))d\tau \bigg ) \textbf{1}_{B_R}(u)d\mu _{s,N}(u) \\&\le \frac{1}{|t|} \int \int _{I_t} \exp \big ( t(p-1) \mathcal {Q}_{s,N}(\Phi _{\tau }^{N}(u))\big ) d\tau \textbf{1}_{B_R}(u)d\mu _{s,N}(u) \\&= \frac{1}{|t|} \int _{I_t} \int \exp \big ( t(p-1) \mathcal {Q}_{s,N}(\Phi _{\tau }^{N}(u))\big ) \textbf{1}_{B_R}(u)d\mu _{s,N}(u) d\tau \\&= \frac{1}{|t|} \int _{I_t} \int \exp \big ( t(p-1) \mathcal {Q}_{s,N}(u)\big ) \textbf{1}_{B_R}(u) f_{\tau }^{N}(u)d\mu _{s,N}(u)d\tau \\&\le \big \Vert e^{|t(p-1)\mathcal {Q}_{s,N}(u)|}\textbf{1}_{B_R}\Vert _{L^{p'}(d\mu _{s,N})} \sup _{\tau \in I_{t}}\Vert f_{\tau }^{N}\textbf{1}_{B_R}\Vert _{L^{p}(d\mu _{s,N})} \\&= \bigg ( \int e^{|tp\mathcal {Q}_{s,N}(u)|}\textbf{1}_{B_R}(u)d\mu _{s,N}(u)\bigg )^{\frac{1}{p'}} \sup _{\tau \in I_{t}}\Vert f_{\tau }^{N}\textbf{1}_{B_R}\Vert _{L^{p}(d\mu _{s,N})}. \end{aligned}$$

Note that for each fixed $\tau \in I_t$, we can repeat this argument to also obtain

$$\begin{aligned} \sup _{\tau \in I_{t}}\Vert f_{\tau }^{N} \textbf{1}_{B_R}\Vert _{L^p (\mu _{s,N})}^{p} \le \bigg ( \int e^{|tp\mathcal {Q}_{s,N}(u)|}\textbf{1}_{B_R}(u)d\mu _{s,N}(u)\bigg )^{\frac{1}{p'}} \sup _{\tau \in I_{t}}\Vert f_{\tau }^{N}\textbf{1}_{B_R}\Vert _{L^{p}(d\mu _{s,N})}, \end{aligned}$$

where we used that $e^{|tp\mathcal {Q}_{s,N}(u)|}$ is increasing in |*t*|. Thus, by Remark (3.2), we find

$$\begin{aligned} \sup _{\tau \in I_{t}}\Vert f_{t}^{N} \textbf{1}_{B_R}\Vert _{L^p (\mu _{s,N})}&\le \bigg (\int e^{|tp\mathcal {Q}_{s,N}(u)|}\textbf{1}_{B_R}(u)d\mu _{s,N}(u) \bigg )^{\frac{1}{p}} \\&= \bigg (\int e^{|tp\mathcal {Q}_{s,N}(u)|\textbf{1}_{B_R}(u)}d\mu _{s,N}(u) -\mu _{s,N}(B_{R}) \bigg )^{\frac{1}{p}} \\&\le \bigg (\int e^{|tp\mathcal {Q}_{s,N}(u)|\textbf{1}_{B_R}(u)}d\mu _{s,N}(u) \bigg )^{\frac{1}{p}}. \end{aligned}$$

The estimate (3.10) now follows from this and Lemma 3.3. □

We now iterate the short-time bounds (3.11) to reach arbitrary times $t\in \mathbb {R}$, namely to times which no longer depend on *R*. The argument here is similar to that in [17]. We point out again that this extra step seems to be necessary due to the restriction on λ in Lemma 3.3 which itself is a symptom of the critical nature of (1.1) and the use of an energy cut-off.

### Proposition 3.5

Let $\frac{1}{2} <s\le 1$, $t\in \mathbb {R}$, and R>0. Then, there exists p(t,R)>1, and constants $C_1 (R), C_2 (R)>0$ such that

3.11 $$\begin{aligned} \sup _{N\in \mathbb {N}} \Vert f_{t}^{N}\textbf{1}_{B_{R}}\Vert _{L^{p(t,R)}(d\mu _{s,N})} \le C_1(R) e^{C_2(R)t}. \end{aligned}$$

The strategy is similar to that of [17, Proposition 4.14]; however, to make this article as self-contained as possible, we provide some of the details below as well as the modifications. In particular, to obtain *p*(*t*, *R*) for general $t\in \mathbb {R}$, we need to more carefully choose the exponents later in the argument as compared to [17].

### Proof

First, we claim that

3.12 $$\begin{aligned} \Vert f_{t+\tau }^{N} \textbf{1}_{B_R}\Vert _{L^r(d\mu _{s,N})} \le \Vert f_{\tau }^{N}\textbf{1}_{B_R}\Vert _{L^p(d\mu _{s,N})} \Vert f_{t}^{N}\textbf{1}_{B_R}\Vert _{L^{\frac{r(p-1)}{p-r}}(d\mu _{s,N})}^{\frac{1}{p'}} \end{aligned}$$

for any $t, \tau \in \mathbb {R}$ and $1\le r<p<\infty$. Let $F:H^{\frac{1}{2}}(\mathbb {T})\rightarrow \mathbb {R}$ be continuous and bounded. Then, by the defining property of the push-forward measure $(\Phi _{t}^{N})_{\#} \mu _s$, the group property of the flows $\Phi _{t}^{N}$, the invariance of $B_{R}$ under the flow, and Hölder’s inequality, we have

3.13 $$\begin{aligned} \bigg |\int F(u) f_{t+\tau }^{N}(u) \textbf{1}_{B_R} d\mu _{s,N}(u)\bigg |&=\bigg | \int F(\Phi _{t+\tau }^{N}(u)) \textbf{1}_{B_R}(\Phi _{t+\tau }^{N}(u)) d\mu _{s,N}\bigg | \nonumber \\&=\bigg | \int F( \Phi ^{N}_{\tau }\circ \Phi _{t}^{N}(u)) \textbf{1}_{B_{R}}(\Phi ^{N}_{\tau }\circ \Phi _{t}^{N}(u)) d\mu _{s,N}\bigg | \nonumber \\&= \bigg |\int F(\Phi _{t}(u))\textbf{1}_{B_{R}}(u) f_{\tau }^{N}(u) d\mu _{s,N} \bigg | \nonumber \\&\le \bigg ( \int |F(\Phi _{t}^{N}(u))|^{p'}\textbf{1}_{B_R}(u)d\mu _{s,N}\bigg )^{\frac{1}{p'}} \Vert f_{\tau }^{N}\textbf{1}_{B_R}\Vert _{L^{p}(d\mu _{s,N})} \nonumber \\&= \bigg ( \int |F(u)|^{p'} f_{t}^{N}(u) \textbf{1}_{B_R}(u)d\mu _{s,N} \bigg )^{\frac{1}{p'}}\Vert f_{\tau }^{N}\textbf{1}_{B_R}\Vert _{L^{p}(d\mu _{s,N})} \nonumber \\&\le \Vert F\Vert _{L^{r'}(d\mu _{s,N})} \Vert f_{t}^{N}\textbf{1}_{B_R}\Vert _{L^{\frac{r(p-1)}{p-r}}(d\mu _{s,N})}^{\frac{1}{p'}}\Vert f_{\tau }^{N}\textbf{1}_{B_R}\Vert _{L^{p}(d\mu _{s,N})}. \end{aligned}$$

Now, (3.12) follows from (3.13) and duality.

We now iterate (3.12) to cover general $t\in \mathbb {R}$. We consider positive times as the argument for negative times is the same. Given $1<r_1<\infty$, let $p>r_1$ be sufficiently large so that

3.14 $$\begin{aligned} \frac{p^2}{2p-1} >r_1 \end{aligned}$$

and choose $\tau _{R}>0$ such that

3.15 $$\begin{aligned} p\tau _{R}=\min (c_0 R^{-\frac{1}{2}},1)=:\Lambda (R), \end{aligned}$$

where $c_0$ is the constant in Lemma 3.3. For $j\in \mathbb {N}$, we define the sequence of times $t_j= j \tau _{R}$ and integrability exponents $r_{j}$ by

$$\begin{aligned} r_{j+1}= \frac{r_{j}p}{p+r_{j}-1} \quad \text {and} \quad r_{j}\vert _{j=1}=r_1. \end{aligned}$$

Note that $r_{j}= \frac{r_{j+1}(p-1)}{p-r_{j+1}}$ and $1<r_{j+1}<r_j$ for all $j\in \mathbb {N}$. In fact, a straightforward argument by induction on $j\in \mathbb {N}$ shows that

3.16 $$\begin{aligned} r_{j} = \frac{r_1(p-1)}{r_1 (p-1)-p(r_1-1)\frac{1}{(p')^{j}}} = \Big [ 1-\tfrac{p'}{r_1'} e^{-j(\log p')}\Big ]^{-1} = \Big [ 1-\tfrac{p'}{r_1'} e^{-\frac{ t_{j}}{\tau _{R}}(\log p')}\Big ]^{-1}. \end{aligned}$$

Thus, (3.12) implies

3.17 $$\begin{aligned} \Vert f_{t_{j+1}}^{N}\textbf{1}_{B_R} \Vert _{L^{r_{j+1}}(d\mu _s)} \le \Vert f_{\tau _{R}}^{N}\textbf{1}_{B_R}\Vert _{L^{p}(d\mu _s)} \Vert f_{t_j}^{N}\textbf{1}_{B_R} \Vert _{L^{r_{j}}(d\mu _s)}^{\frac{1}{p'}}. \end{aligned}$$

This sets up a recursive inequality for which we get

3.18 $$\begin{aligned} \Vert f_{t_{j}}^{N}\textbf{1}_{B_R} \Vert _{L^{r_{j}}(d\mu _s)} \le \Vert f_{\tau _{R}}^{N}\textbf{1}_{B_R}\Vert _{L^{p}(d\mu _s)}^{\sum _{k=0}^{j}(p')^{-k}} \le \max ( 1, \Vert f_{\tau _{R}}^{N} \textbf{1}_{B_R}\Vert _{L^{p}(d\mu _s)})^{j}. \end{aligned}$$

Therefore, by Lemma 3.4 (with $\lambda = \Lambda (R)\le 1$) and (3.15),

3.19 $$\begin{aligned} \sup _{N\in \mathbb {N}}\Vert f_{t_{j}}^{N}\textbf{1}_{B_R} \Vert _{L^{r_{j}}(d\mu _s)} \le \exp \big (\widetilde{C}(R){\tfrac{t_j}{\tau _R}}\big ), \end{aligned}$$

where $$\widetilde{C}(R):=C(R)\Lambda (R)$$.

We now obtain bounds for general t>0. As we need to account for a further deterioration in the integrability exponent, and in view of (3.16), we define $q:[0,\infty )\rightarrow (1,\infty )$ by

3.20 $$\begin{aligned} q(t) := \Big [ 1-\tfrac{p'}{r_1'} \exp (-\tfrac{2t}{\tau _{R}}(\log p'))\Big ]^{-1}. \end{aligned}$$

We see that *q*(*t*) is monotonically decreasing, $q(0) = \frac{r_1 (p-1)}{p-r_1}<p$ by (3.14), and by (3.15),

$$\begin{aligned} q(t) t \le q(0)t \le q(0) \tau _{R} <\Lambda (R) \end{aligned}$$

for all $0\le t\le \tau _{R}$. Therefore, we may directly apply Lemma 3.4 to obtain

3.21 $$\begin{aligned} \sup _{N\in \mathbb {N}} \Vert f_{t}^{N}\textbf{1}_{B_R}\Vert _{L^{q(t)}(d\mu _{s,N})} \le e^{C(R)|t|q(t)}. \end{aligned}$$

Now, assume that $t>\tau _{R}$ and such that there is $j_0\in \mathbb {N}$ (which depends on *t* and $\tau _R$) such that $j_0 \tau _{R}<t <(j_0 +1)\tau _{R}$. By repeating the argument which derived (3.12), we obtain

3.22 $$\begin{aligned} \Vert f_{t}^{N}\textbf{1}_{B_R}\Vert _{L^r (d\mu _{s,N})} \le \Vert f_{t-j_0 \tau _{R}}^{N}\textbf{1}_{B_R}\Vert _{L^{p}(d\mu _{s,N})} \Vert f_{j_0 \tau _{R}}^{N} \textbf{1}_{B_R}\Vert _{L^{ \frac{r(p-1)}{p-r}}(d\mu _{s,N})}^{\frac{1}{p'}} \end{aligned}$$

for any $1\le r<p<\infty$. We apply (3.22) with r=q(t). Since $j_0<\frac{t}{\tau _{R}}<j_0+1$, it follows from (3.20) and (3.16) that

$$\begin{aligned} \tfrac{q(t)(p-1)}{p-q(t)}=q\big (t-\tfrac{\tau _R}{2}\big ) \le r_{j_0}. \end{aligned}$$

Thus, by (3.22), (3.21), Hölder’s inequality, and (3.19), we obtain

$$\begin{aligned} \Vert f_{t}^{N}\textbf{1}_{B_R}\Vert _{L^{q(t)}(d\mu _{s,N})}&\le e^{C(R) \tau _{R}q(\tau _R)} \Vert f_{j_0 \tau _{R}}\textbf{1}_{B_R}\Vert _{L^{q(t-\frac{\tau _R}{2})}(d\mu _{s,N})}^{\frac{1}{p'}} \\&\le e^{C(R) \tau _{R}q(0) } \Vert f_{j_0 \tau _{R}}\textbf{1}_{B_R}\Vert _{L^{r_{j_0}}(d\mu _{s,N})}^{\frac{1}{p'}} \\&\le e^{C(R) \tau _{R}q(0) } e^{\widetilde{C}(R)\frac{t_{j_0}}{p'\tau _{R}}} \\&\le \exp \big (\widetilde{C}(R)( 1+ \tfrac{p-1}{\Lambda (R)}t)\big ), \end{aligned}$$

uniformly in $N\in \mathbb {N}$. Combining this with (3.19) and (3.21) completes the proof. □

We now move onto the proof of Theorem 1.2.

### Proof of Theorem 1.2

Let $\frac{1}{2}<\sigma <s$. By the global well-posedness for (1.1) and (3.1) in $H^{\sigma }(\mathbb {T})$ [29], and a standard argument, for each $u_0\in H^{\sigma }$, we have

3.23 $$\begin{aligned} \lim _{N\rightarrow \infty }\Vert \Phi _{t}(u_0)-\Phi _{t}^{N}(\Pi _{\le N}u_0)\Vert _{C([-T,T];H^{\sigma '})}=0 \end{aligned}$$

for every $\sigma '<\sigma$ and T>0.

Fix $t\in \mathbb {R}$ and R>0. Then, with p=p(t,R)>1 as in Proposition 3.5, (3.11) implies that there exists $f_{t,R}\in L^{p}(d\mu _s)$ such that, up to a subsequence, $(f_{t}^{N}\textbf{1}_{B_R})\circ \Pi _{\le N}$ converges weakly to $f_{t,R}$ in $L^p(d\mu _s)$ as $N\rightarrow \infty$.

We now show that $f_{t,R}$ is supported on the ball $B_{R}$ in $H^{1/2}_0(\mathbb {T})$. Let δ>0. By the weak convergence of $(f_{t}^{N}\textbf{1}_{B_R})\circ \Pi _{\le N}$, independence of $\Pi _{\le N}u$ and $\Pi _{>N}u$ for $N\in \mathbb {N}$, and (2.1), we have

$$\begin{aligned} \int&f_{t,R}(u)\textbf{1}_{B_{R+\delta }^{c}}(u)d\mu _{s}(u) \\&= \lim _{N\rightarrow \infty } \int f_{t}^{N}(\Pi _{\le N}u) \textbf{1}_{B_R}(\Pi _{\le N}u) \textbf{1}_{B_{R+\delta }^{c}}(u)d\mu _{s} \\&= \lim _{N\rightarrow \infty } \int f_{t}^{N}(u_N) \textbf{1}_{B_R}(u_N) \int \textbf{1}_{B_{R+\delta }^{c}}(u_N +u_{N}^{\perp }) d\mu _{s,N}^{\perp } d\mu _{s,N} \\&= \lim _{N\rightarrow \infty } \int f_{t}^{N}(u_N) \textbf{1}_{B_R}(u_N) \mu _{s,N}^{\perp }\bigg ( \Vert u_{N}^{\perp }\Vert _{H^{1/2}_{0}}^2 > (R+\delta )^{2} -\Vert u_N\Vert _{H^{1/2}_{0}}^{2} \bigg ) d\mu _{s,N} \\&\le C \limsup _{N\rightarrow \infty } \frac{1}{\delta RN^{2s-1}} \int f_{t}^{N}(u_N) \textbf{1}_{B_R}(u_N) d\mu _{s,N} \\&\le C \limsup _{N\rightarrow \infty } \frac{1}{\delta RN^{2s-1}} =0. \end{aligned}$$

Thus,

$$\begin{aligned} \int f_{t,R}(u) \textbf{1}_{B_{R+\delta }^{c}}(u) d\mu _s =0 \end{aligned}$$

for every δ>0, and so by the monotone convergence theorem, we conclude that

3.24 $$\begin{aligned} f_{t,R} \textbf{1}_{B_{R}^{c}}=0 \quad \mu _s \,\,\text {a.e.} \end{aligned}$$

Now, we show that $f_{t,R}$ is the Radon–Nikodym derivative of $(\Phi _{t})_{\#}\mu _{s,R}$ with respect to $\mu _{s,R}$. Assuming this for now, we get $(\Phi _{t})_{\#}\mu _{s,R} \ll \mu _{s,R}$. By taking $R\rightarrow \infty$, this implies $(\Phi _{t})_{\#}\mu _{s} \ll \mu _{s}$. Let $F:H^{1/2}_{0}(\mathbb {T})\rightarrow \mathbb {R}$ be continuous, bounded, and non-negative. By the convergence in (3.23) and the dominated convergence theorem

3.25 $$\begin{aligned} \lim _{N\rightarrow \infty } \int |F(\Phi _{t}(u)) -F(\Phi ^{N}_{t}(\Pi _{\le N}u))| \textbf{1}_{B_R}(\Pi _{\le N}u) d\mu _s = 0. \end{aligned}$$

Similarly, by (3.11) and the dominated convergence theorem,

3.26 $$\begin{aligned} \lim _{N\rightarrow \infty } \int |F(u) -F(\Pi _{\le N}u)| f_{t}^{N}(\Pi _{\le N}u)\textbf{1}_{B_R}(\Pi _{\le N}u) d\mu _s = 0. \end{aligned}$$

Then, by monotone convergence, (3.25), (3.26) and (3.24), we have

$$\begin{aligned} \int F(u) d(\Phi _{t})_{\#}\mu _{s,R}&= \int F(\Phi _{t}(u)) \textbf{1}_{B_R}(u) d\mu _s \\&= \lim _{N\rightarrow \infty } \int F(\Phi _{t}(u)) \textbf{1}_{B_R}(\Pi _{\le N}u) d\mu _s \\&= \lim _{N\rightarrow \infty } \int F(\Phi ^{N}_{t}(\Pi _{\le N}u))\textbf{1}_{B_R}(\Pi _{\le N}u) d\mu _s \\&= \lim _{N\rightarrow \infty } \int F(\Phi ^{N}_{t}(\Pi _{\le N}u))\textbf{1}_{B_R}(\Pi _{\le N}\Phi _{t}^{N}(\Pi _{\le N}u)) d\mu _s\\&= \lim _{N\rightarrow \infty } \int F(\Pi _{\le N}u) f_{t}^{N}(\Pi _{\le N}u) \textbf{1}_{B_R}(\Pi _{\le N}u)d\mu _{s} \\&= \lim _{N\rightarrow \infty } \int F(u) f_{t}^{N}(\Pi _{\le N}u) \textbf{1}_{B_R}(\Pi _{\le N}u)d\mu _{s} \\&= \int F(u) f_{t,R}(u) d\mu _s \\&= \int F(u) f_{t,R}(u) d\mu _{s,R}. \end{aligned}$$

As this identity holds for all such *F*, we obtain that $d(\Phi _{t})_{\#}\mu _{s,R} = f_{t,R} d\mu _{s,R}$. This completes the proof of Theorem 1.2. □

## Proof of Lemma 3.3

We need exponential integrability of $\mathcal {Q}_{s,N}$, uniformly in *N* and locally on the support of $\mu _{s,N}$. Using the simplified variational formula [16, Lemma 2.6], we have

4.1 $$\begin{aligned} \begin{aligned} \log \int&\exp \big ( \lambda |\mathcal {Q}_{s,N}(u)| \textbf{1}_{B_{R}}(u)\big ) d\mu _{s,N} \\&\le \mathbb {E}\bigg [ \sup _{V\in H^{s+1/2}}\Big \{ \lambda |\mathcal {Q}_{s,N}(Y_{N}+V_{N})|\textbf{1}_{B_R}(Y_{N}+V_{N}) -\tfrac{1}{2} \Vert V_N\Vert _{H^{s+1/2}}^{2} \Big \} \bigg ], \end{aligned} \end{aligned}$$

where *Y* is distributed according to $\mu _{s,N}$, $Y_{N}:=\textbf{P}_{\le N}Y$, and $V_{N}:=\textbf{P}_{\le N}V$. For $Y_{N}+V_{N}\in B_{R}$, the triangle inequality implies

4.2 $$\begin{aligned} \Vert V_{N}\Vert _{H^{1/2}} \le R^{1/2}+ \Vert Y_{N}\Vert _{H^{1/2}}. \end{aligned}$$

The goal is to show that the right-hand side of (4.1) is finite, uniformly in $N\in \mathbb {N}$. More precisely, we will show that

4.3 $$\begin{aligned} \text {RHS} (4.1) \le C (\lambda + \lambda ^{c_1(s)+1}) (1+R^{c_2(s)}) \le C \max (\lambda ,\lambda ^{c_1(s)}) [1+R^{c_2(s)}], \end{aligned}$$

provided that $\lambda R^{\frac{1}{2}}\ll 1$,^2^ and for some $c_1(s), c_2(s)>0$ and C>0 which do not depend on *N*. Here, $c_1(s)$ and $c_2(s)$ are in principle computable, with the largest power $c_2$ coming from (4.8). Then, (3.9) is obtained from combining (4.1) and (4.3) and taking the exponential of both sides, which leads to $C(R) =C[1+R^{c_2 (s)}]$ and $A=c_{1}(s)$.

The key is to control the *V*-factors by the negative terms $\frac{1}{2} \Vert V_N\Vert _{H^{s+1/2}}^{2}$, as well as leveraging the cut-off $\textbf{1}_{B_R}$ to control the potential higher homogeneity from the terms in $\mathcal {Q}_{s,N}(Y_N +V_N)$ which contain exactly three *V* inputs. To do this, we need to decompose the operator $\mathcal {Q}_{s,N}$ as in (3.5) and then further decompose each of $\mathcal {Q}_{s,N}^{(j)}$, j=1,2 as in (3.6) and (3.7) into a certain *finite* number of parts $L\in \mathbb {N}$; namely,

$$\begin{aligned} |\mathcal {Q}_{s,N}(Y_N+V_N)| \lesssim \sum _{j=1}^{2}\sum _{\ell =1}^{L} |\mathcal {Q}^{(j)}_{s,N,\ell }(Y_N+V_N)| \end{aligned}$$

for some operators $\{\mathcal {Q}^{(j)}_{s,N,\ell }\}_{\ell =1}^{L}$, j=1,2, and control their contributions to (4.1) separately.

In order to use the negative terms in $\frac{1}{2} \Vert V_N\Vert _{H^{s+1/2}}^{2}$ for each of these $\{\mathcal {Q}_{s,N,\ell }\}_{\ell =1}^{L}$, we use

$$\begin{aligned} \text {RHS} (4.1) \le \sum _{j=1}^{2}\sum _{\ell =1}^{L} \mathbb {E}\Big [ \sup _{V\in H^{s+1/2}}\Big \{ \lambda |\mathcal {Q}^{(j)}_{s,N,\ell }(Y_N+V_N)|\textbf{1}_{B_R}(Y_N+V_N) -\tfrac{1}{4L} \Vert V_N\Vert _{H^{s+1/2}}^{2} \big \} \Big ]. \end{aligned}$$

It then suffices to control each of the summands. To be concrete, it will be more than enough in the following to take L=100.

In the following sections, to simplify the notation, we write *Y* and *V* in place of $Y_N$ and $V_N$, respectively.

### Contribution from $\mathcal {Q}^{(1)}_{s,N}$

In view of Lemma 3.1, we make the following dyadic decompositions:

4.4 $$\begin{aligned} \mathcal {Q}^{(1)}_{s,N}(u)&= \sum _{ \begin{array}{c} N_1, N_2, N_3 \in 2^{\mathbb {N}_0} \\ N_{1} \sim N_{2} \sim N_{3} \end{array}}\mathcal {Q}^{(1)}_{s,N}( \textbf{P}_{N_1}u, \textbf{P}_{N_2}u, \textbf{P}_{N_3}u) \end{aligned}$$

4.5 $$\begin{aligned}&+ \sum _{ \begin{array}{c} N_1, N_2, N_3 \in 2^{\mathbb {N}_0} \\ N_{(2)}\gg N_{(3)} \end{array}}\mathcal {Q}^{(1)}_{s,N}( \textbf{P}_{N_1}u, \textbf{P}_{N_2}u, \textbf{P}_{N_3}u). \end{aligned}$$

We first consider the contribution from (4.5). By symmetry, we may assume that $N_1 \ge N_2 \ge N_3$. Then, with u=Y+V, we have the following unique cases to consider:

$$\begin{aligned}&(Y_{N_1},Y_{N_2},Y_{N_3}), \,\,\,(Y_{N_1},Y_{N_2},V_{N_3}), \,\,\, (Y_{N_1},V_{N_2},Y_{N_3}), \,\,\,\\&(Y_{N_1}, V_{N_2},V_{N_3}), \,\,\, (V_{N_1},V_{N_2},Y_{N_3}+V_{N_3}). \end{aligned}$$

In these cases, by Lemma 3.1, we always have $|\Psi _{s}(\overline{n})|\lesssim N_{1}^{2s} N_3$.

$${\underline{{\textbf {Case 1:}}\,\, (Y_{N_1},Y_{N_2},Y_{N_3})}}$$

For this case, we need to use the gain coming from random oscillations. We have

$$\begin{aligned} \mathbb {E}\big [ |\mathcal {Q}^{(1)}_{s,N}(Y_{N_1}, Y_{N_2}, Y_{N_3})|^{2}\big ]&= \mathbb {E}\bigg [ \bigg | \sum _{ n_{123}=0} \Psi _{s}(\overline{n}) \prod _{j=1}^{3} \phi _{N_j}(n_j) \widehat{Y}(n_j) \bigg |^{2} \bigg ] \\&= \sum _{ \begin{array}{c} n_{123}=0 \\ m_{123}=0 \end{array}} \Psi _{s}(\overline{n}) \Psi _{s}(\overline{m})\prod _{j=1}^{3} \frac{ \phi _{N_j}(n_j) \phi _{N_j}(m_j) }{ |n_j|^{s+1/2}|m_j|^{s+1/2}} \\&\quad \mathbb {E}[ g_{n_1}g_{n_2}g_{n_3}\overline{g_{m_1}g_{m_2}g_{m_3}}]. \end{aligned}$$

We use Isserlis theorem to control the expectation and exploit the independence structure. As all the individual frequencies are nonzero, we cannot have any self-interactions of the form $$n_{j}+n_{j'}=0$$ for $j\neq j^{\prime }$. Moreover, since $$N_1, N_2 \gg N_3$$, we must have that $$n_3= m_3$$ and thus $$(n_1,n_2)=(m_1,m_2)$$ or $$(n_1,n_2)=(m_2,m_1)$$. Both of these cases yield the same contribution, so we then have

$$\begin{aligned}&\lesssim \sum _{ \begin{array}{c} n_{123} =0 \\ |n_j| \sim N_j, j=1,2,3 \end{array}} |\Psi _{s}(\overline{n})|^{2} (|n_1||n_2||n_3|)^{-2s-1}\\&\lesssim N_{1}^{4s} N_{3}^{2} N_{1}^{-4s-2}N_{3}^{-2s-1} N_{1}N_{3} \lesssim N_{1}^{-1}N_{3} N_{3}^{1-2s} \end{aligned}$$

By first summing in $N_1$ over $N_1 \gg N_3$ and using that $s>\frac{1}{2}$ to sum in $N_3$, this contribution to (4.1) is thus controlled.

$${\underline{{\textbf {Case 2:}}\,\, (Y_{N_1},Y_{N_2},V_{N_3})}}$$

We write

4.6 $$\begin{aligned} \mathcal {Q}^{(1)}_{s,N}( Y_{N_1}, Y_{N_2}, V_{N_3}) = \sum _{n_3} |n_3|^{s+\frac{1}{2}} \widehat{V}(n_3) \phi _{N_3}(n_3)|n_3|^{-s-\frac{1}{2}} K^{(1)}_{N_1 N_2 N_3}(n_3), \end{aligned}$$

where

4.7 $$\begin{aligned} K^{(1)}_{N_1 N_2 N_3}(n_3) : =\sum _{n_1, n_2} \textbf{1}_{\{n_1+n_2+n_3=0\}} \Psi _{s}(\overline{n}) \phi _{N_1}(n_1) \phi _{N_2}(n_2) \widehat{Y}(n_1) \widehat{Y}(n_2). \end{aligned}$$

Let $0<\delta <\min (s-\frac{1}{2}, \frac{1}{2})$ and $\varepsilon =\varepsilon (s,\delta ,L)>0$ such that

$$\begin{aligned} 2L \varepsilon \sum _{ \begin{array}{c} N_1, N_2, N_3 \in 2^{\mathbb {N}_0} \\ N_1 \sim N_{2}\gg N_{3} \end{array}}N_{1}^{-2\delta } \le \frac{1}{4L}. \end{aligned}$$

Then, by Cauchy–Schwarz and Cauchy’s inequality, we have

$$\begin{aligned} \lambda |\mathcal {Q}^{(1)}_{s,N}( Y_{N_1}, Y_{N_2}, V_{N_3}) |&\le \varepsilon N_{1}^{-2\delta } \Vert V\Vert _{H^{s+\frac{1}{2}}}^{2} \\&\quad + \lambda ^2 C_{\varepsilon }N_{1}^{2\delta }\sum _{n_3} |\phi _{N_3}(n_3)|^2 |n_3|^{-2s-1} |K^{(1)}_{N_1 N_2 N_3}(n_3)|^{2}. \end{aligned}$$

It remains to control the expected value of the second term. As in Case 1, we have

$$\begin{aligned} \mathbb {E}\bigg [ N_{1}^{2\delta } \sum _{n_3}&|\phi _{N_3}(n_3)|^2 |n_3|^{-2s-1} |K^{(1)}_{N_1 N_2 N_3}(n_3)|^{2} \bigg ]\\&\sim N_{1}^{2\delta } N_{3}^{-2s-1} \sum _{|n_3|\sim N_3} \sum _{ \begin{array}{c} n_1,n_2 \\ n_1+n_2+n_3=0 \end{array}} \frac{|\Psi _{s}(\overline{n}) \phi _{N_1}(n_1) \phi _{N_2}(n_2)|^2 }{(|n_1||n_2|)^{2s+1}} \\&\lesssim N_{1}^{2\delta } N_{3}^{-2s-1} \cdot N_{3}N_{1} \cdot N_{1}^{-4s-2} \cdot N_{1}^{4s}N_{3}^{2} \sim \bigg ( \frac{N_3}{N_1}\bigg )^{1-2\delta } N_{3}^{-2s+1+2\delta }. \end{aligned}$$

This is summable, first over $N_1\gg N_3$, and then over $N_3$ since $s>\frac{1}{2}$.

$${\underline{{\textbf {Case 3:}}\,\, (Y_{N_1},V_{N_2},Y_{N_3})}}$$

In this case, we proceed similarly to Case 2, writing

$$\begin{aligned} \mathcal {Q}^{(1)}_{s,N}( Y_{N_1}, V_{N_2}, Y_{N_3}) = \sum _{n_2} |n_2|^{s+\frac{1}{2}} \widehat{V}(n_2) \phi _{N_2}(n_2)|n_2|^{-s-\frac{1}{2}} K^{(2)}_{N_1 N_2 N_3}(n_2), \end{aligned}$$

where

$$\begin{aligned} K^{(2)}_{N_1 N_2 N_3}(n_2) : = \sum _{n_1, n_3} \textbf{1}_{\{ n_{123}=0\}} \Psi _{s}(\overline{n}) \phi _{N_1}(n_1)\phi _{N_3}(n_3) \widehat{Y}(n_1) \widehat{Y}(n_3). \end{aligned}$$

Note that $\mathbb {E}[g_{n_1}g_{n_3}\overline{g_{m_1}g_{m_3}}]= \delta _{n_1m_1}\delta _{n_3 m_3}$ since $|n_1|\gg |n_3|$. Thus, we have

$$\begin{aligned}&\mathbb {E}\bigg [ N_{2}^{2\delta } \sum _{n_2} |\phi _{N_2}(n_2)|^2 |n_2|^{-2s-1} |K^{(2)}_{N_1 N_2 N_3}(n_2)|^{2} \bigg ] \\&\sim N_2^{2\delta -2s-1} \cdot N_{1}^{4s}N_{3}^{2} \cdot N_{1}N_{3}\cdot (N_{1}N_{3})^{-2s-1} \\&\lesssim \bigg ( \frac{N_3}{N_1}\bigg )^{1-2\delta } N_{3}^{-2s+1+2\delta }. \end{aligned}$$

We then sum as in Case 2.

$${\underline{{\textbf {Case 4:}}\,\, (Y_{N_1},V_{N_2},V_{N_3})}}$$

A simple Cauchy–Schwarz argument, that uses only the regularity of $Y_{N_1}$ and not its Gaussian structure, suffices to control this contribution when s<1 but fails when s≥1. In the following, we present a unified approach that deals with all $s>\frac{1}{2}$, which does use the explicit structure of $Y_{N_1}$. Let $0<\delta <\frac{1}{4} (s-\frac{1}{2})$, $\varepsilon =\varepsilon (s,\delta )>0$ such that

$$\begin{aligned} 2L \varepsilon \sum _{ \begin{array}{c} N_1, N_2, N_3 \in 2^{\mathbb {N}_0} \\ N_1 \sim N_{2}\gg N_{3} \end{array}}N_{1}^{-\frac{2\delta s}{2s- \delta }} \le \frac{1}{4L}, \end{aligned}$$

and define the random variable

$$\begin{aligned} B_{N_1}(\omega ) : = \bigg ( \sum _{n_1 \in \mathbb {Z}_{*}} |\phi _{N_1}(n_1)|^{2} |\widehat{Y}(n_1)|^{2} \bigg )^{1/2}. \end{aligned}$$

By Cauchy–Schwarz, interpolation, (4.2), and Young’s inequality, we have

4.8 $$\begin{aligned} \begin{aligned} \lambda&|\mathcal {Q}^{(1)}_{s,N}( Y_{N_1}, V_{N_2}, Y_{N_3})| \\&\lesssim \lambda N_{1}^{2s} N_{3} \sum _{n_{123}=0} |\widehat{V}_{N_1}(n_1)| |\widehat{Y}_{N_2}(n_2)| |\widehat{V}_{N_3}(n_3)| \\&\lesssim \lambda N_{1}^{2s}N_{3} \cdot N_{2}^{-s-\frac{1}{2}} N_{3}^{\frac{1}{2}} \cdot N_{3}^{-s-\frac{1}{2} +\delta } B_{N_1}(\omega ) \Vert V_{N_2}\Vert _{H^{s+\frac{1}{2}}} \Vert V_{N_3}\Vert _{H^{s+\frac{1}{2} -\delta }} \\&\lesssim \lambda N_{1}^{s-\frac{1}{2}}N_{3}^{1-s+\delta } B_{N_1}(\omega ) \Vert V_{N_2}\Vert _{H^{s+\frac{1}{2}}} \Vert V_{N_3}\Vert _{H^{s+\frac{1}{2}}}^{1-\frac{\delta }{s}} \Vert V_{N_2}\Vert _{H^{\frac{1}{2}}}^{\frac{\delta }{s}} \\&\lesssim \lambda N_{1}^{s-\frac{1}{2}}N_{3}^{1-s+\delta }B_{N_1}(\omega ) (R^{\frac{1}{2}}+\Vert Y_{N_3}\Vert _{H^{\frac{1}{2}}})^{\frac{\delta }{s}} \Vert V_N\Vert _{H^{s+\frac{1}{2}}}^{2-\frac{\delta }{s}} \\&\lesssim \lambda ^{\frac{2s}{\delta }}C_{\varepsilon ,\delta } \big [N_{1}^{s-\frac{1}{2}+\delta }N_{3}^{1-s+\delta }B_{N_1}(\omega ) (R^{\frac{1}{2}}+\Vert Y_{N_3}\Vert _{H^{\frac{1}{2}}})^{\frac{\delta }{s}} \big ]^{\frac{2s}{\delta }} + \varepsilon N_{1}^{-\frac{2\delta s}{2s-\delta }} \Vert V_N\Vert _{H^{s+\frac{1}{2}}}^{2}. \end{aligned} \end{aligned}$$

It remains to control the first term under after taking an expectation. For any finite p≥2, Minkowski’s inequality implies

4.9 $$\begin{aligned} \mathbb {E}\big [ B_{N_1}(\omega )^{p}\big ]^{\frac{1}{p}} \lesssim \bigg ( \sum _{n_1 \in \mathbb {Z}_{*}} |\phi _{N_1}(n_1)|^{2} |n_1|^{-2s-1} \mathbb {E}[|g_{n_1}|^{p}]^{\frac{2}{p}}\bigg )^{\frac{1}{2}} \lesssim _p N_{1}^{-s}. \end{aligned}$$

Thus, by Cauchy–Schwarz and (4.9), we have

$$\begin{aligned} \mathbb {E}\bigg [&\sum _{ \begin{array}{c} N_1, N_2, N_3 \in 2^{\mathbb {N}_0} \\ N_1 \sim N_{2}\gg N_{3} \end{array}} \big [N_{1}^{s-\frac{1}{2}+\delta }N_{3}^{1-s+\delta }B_{N_1}(\omega ) (R^{\frac{1}{2}}+\Vert Y_{N_3}\Vert _{H^{\frac{1}{2}}})^{\frac{\delta }{s}} \big ]^{\frac{2s}{\delta }}\bigg ]\\&= \sum _{ \begin{array}{c} N_1, N_2, N_3 \in 2^{\mathbb {N}_0} \\ N_1 \sim N_{2}\gg N_{3} \end{array}} \big (N_{1}^{s-\frac{1}{2}+\delta }N_{3}^{1-s+\delta } \big )^{\frac{2s}{\delta }} \mathbb {E}\Big [ B_{N_1}(\omega )^{\frac{2s}{\delta }} (R^{\frac{1}{2}}+\Vert Y_{N_3}\Vert _{H^{1/2}})^{2}\Big ]\\&\lesssim \sum _{ \begin{array}{c} N_1, N_2, N_3 \in 2^{\mathbb {N}_0} \\ N_1 \sim N_{2}\gg N_{3} \end{array}} \big (N_{1}^{s-\frac{1}{2}+\delta }N_{3}^{1-s+\delta } \big )^{\frac{2s}{\delta }} \mathbb {E}\big [ B_{N_1}(\omega )^{\frac{4s}{\delta }}\big ]^{\frac{1}{2}} \mathbb {E}[ (R^{\frac{1}{2}}+\Vert Y_{N_3}\Vert _{H^{1/2}})^{4}]^{\frac{1}{2}} \\&\lesssim _{R} \sum _{ \begin{array}{c} N_1, N_2, N_3 \in 2^{\mathbb {N}_0} \\ N_1 \sim N_{2}\gg N_{3} \end{array}} \big (N_{1}^{s-\frac{1}{2}+\delta }N_{3}^{1-s+\delta } N_{1}^{-s} \big )^{\frac{2s}{\delta }}\\&\lesssim _{R} \sum _{ \begin{array}{c} N_1, N_2, N_3 \in 2^{\mathbb {N}_0} \\ N_1 \sim N_{2}\gg N_{3} \end{array}} \Big ( \frac{N_3}{N_1} \Big )^{\frac{2s}{\delta }(\frac{1}{2}-\delta )} N_{3}^{(\frac{1}{2}-s+2\delta ) \frac{2s}{\delta }} \end{aligned}$$

which is summable, first in $N_1\gg N_3$ and using that $s>\frac{1}{2}$ and δ is sufficiently small.

$${\underline{{\textbf {Case 5:}}\,\, (V_{N_1},V_{N_2},Y_{N_3}+V_{N_3})}}$$

By Cauchy–Schwarz, we have

$$\begin{aligned} |\mathcal {Q}^{(1)}_{s,N}&( V_{N_1}, V_{N_2}, Y_{N_3}+V_{N_3})| \\&\lesssim N_{1}^{2s}N_{3}\bigg ( \sum _{ \begin{array}{c} n_{123}=0 \\ |n_j|\sim N_j \end{array}} |\widehat{V}_{N_1}(n_1)|^{2} \bigg )^{\frac{1}{2}}\bigg ( \sum _{ \begin{array}{c} n_{123}=0 \\ |n_j|\sim N_j \end{array}} |\widehat{V}_{N_2}(n_2)|^{2}|\mathcal {F}\{ Y_{N_3}+V_{N_3}\}(n_3)|^2 \bigg )^{\frac{1}{2}} \\&\lesssim N_{1}^{-1} N_{3} \Vert V_{N_1}\Vert _{H^{s+\frac{1}{2}}} \Vert V_{N_2}\Vert _{H^{s+\frac{1}{2}}} \Vert Y_{N_3}+V_{N_3}\Vert _{H^{\frac{1}{2}}}. \end{aligned}$$

We can handle the $N_3$ summation using $\sum _{N_3 \ll N_1} N_{1}^{-1}N_{3}\lesssim 1$, and then the summations over $N_1$ and $N_2$ since

4.10 $$\begin{aligned} \sum _{N_1 \sim N_2} \Vert V_{N_1}\Vert _{H^{s+\frac{1}{2}}} \Vert V_{N_2}\Vert _{H^{s+\frac{1}{2}}} \lesssim \sum _{N_1} \Vert V_{N_1}\Vert _{H^{s+\frac{1}{2}}}\Vert \widetilde{\textbf{P}}_{N_1} V_{N_1}\Vert _{H^{s+\frac{1}{2}}}\lesssim \Vert V\Vert _{H^{s+\frac{1}{2}}}^{2}. \end{aligned}$$

Thus, for $Y+V\in B_{R}$, there is a C>0 independent of $N\in \mathbb {N}$ and R>0, such that

4.11 $$\begin{aligned} \sum _{ \begin{array}{c} N_1, N_2, N_3 \in 2^{\mathbb {N}_0} \\ N_1 \sim N_{2}\gg N_{3} \end{array}} \lambda |\mathcal {Q}^{(1)}_{s,N}( V_{N_1}, V_{N_2}, Y_{N_3}+V_{N_3})| \le C \lambda R^{\frac{1}{2}} \Vert V\Vert _{H^{s+\frac{1}{2}}}^{2}. \end{aligned}$$

Thus,

$$\begin{aligned} \mathbb {E}\Big [ \sup _{V\in H^{s+1/2}} \Big \{ C \lambda R^{\frac{1}{2}} \Vert V\Vert _{H^{s+1/2}}^{2} - \tfrac{1}{4L} \Vert V\Vert _{H^{s+1/2}}^{2} \Big \} \Big ] \le 0 \end{aligned}$$

provided that we choose λ>0 so that

4.12 $$\begin{aligned} C \lambda R^{\frac{1}{2}} \le \frac{1}{4L}. \end{aligned}$$

This completes the estimates for (4.5). We move onto (4.4), where all the frequencies are similar. In this case, we always have $|\Psi _{s}(\overline{n})|\lesssim N_{1}^{2s+1}$. By symmetry, we only have three cases depending on how many *Y* factors there are.

$${\underline{{\textbf {Case 1:}}\,\, (V_{N_1},V_{N_2},Y_{N_3}+V_{N_3})}}$$

This contribution can be handled exactly as in Case 5 when we controlled (4.5), and we obtain

$$\begin{aligned}&\sum _{ \begin{array}{c} N_1, N_2, N_3 \in 2^{\mathbb {N}_0} \\ N_1 \sim N_{2}\sim N_{3} \end{array}} |\mathcal {Q}^{(1)}_{s,N}( V_{N_1}, V_{N_2}, Y_{N_3}+V_{N_3})| \\&\lesssim \sum _{ \begin{array}{c} N_1, N_2, N_3 \in 2^{\mathbb {N}_0} \\ N_1 \sim N_{2}\sim N_{3} \end{array}} \Vert V_{N_1}\Vert _{H^{s+\frac{1}{2}}} \Vert V_{N_2}\Vert _{H^{s+\frac{1}{2}}} \Vert Y_{N_3}+V_{N_3}\Vert _{H^{\frac{1}{2}}} \\&\lesssim \sum _{N_1} \Vert V_{N_1}\Vert _{H^{s+\frac{1}{2}}} \sum _{N_2 \sim N_3} \Vert V_{N_2}\Vert _{H^{s+\frac{1}{2}}} \Vert Y_{N_3}+V_{N_3}\Vert _{H^{\frac{1}{2}}} \\&\lesssim \sum _{N_1} \Vert V_{N_1}\Vert _{H^{s+\frac{1}{2}}} \sum _{N_2 \sim N_1} \Vert V_{N_2}\Vert _{H^{s+\frac{1}{2}}} \Vert \widetilde{\textbf{P}}_{N_2}( Y_{N_2}+V_{N_2})\Vert _{H^{\frac{1}{2}}} \\&\lesssim R^{\frac{1}{2}} \sum _{N_1\sim N_2}\Vert V_{N_1}\Vert _{H^{s+\frac{1}{2}}}\Vert V_{N_2}\Vert _{H^{s+\frac{1}{2}}} \\&\lesssim R^{\frac{1}{2}} \Vert V\Vert _{H^{s+\frac{1}{2}}}^{2}, \end{aligned}$$

which is the same bound as in (4.11).

**Case 2:** $$(Y_{N_1},V_{N_2},Y_{N_3})$$

We argue exactly as in Case 3 when we handled (4.5). With $K^{(2)}_{N_1 N_2N_3}$ chosen analogously to (4.7) in (4.6), when $N_1 \sim N_2\sim N_3$, we obtain

$$\begin{aligned} \mathbb {E}\bigg [ N_{2}^{2\delta } \sum _{n_2} |\phi _{N_2}(n_2)|^2 |n_2|^{-2s-1} |K^{(2)}_{N_1 N_2 N_3}(n_2)|^{2} \bigg ] \lesssim N_{1}^{-2s+1+2\delta } \end{aligned}$$

which is summable as $s>\frac{1}{2}$ and we may choose δ>0 sufficiently small.

**Case 3:** $$(Y_{N_1},Y_{N_2},Y_{N_3})$$

We compute

$$\begin{aligned} \mathbb {E}\big [ |\mathcal {Q}^{(1)}_{s,N}(Y_{N_1}, Y_{N_2}, Y_{N_3})|^{2}\big ]&\lesssim \sum _{ \begin{array}{c} n_{123} =0 \\ |n_j| \sim N_j, j=1,2,3 \end{array}} |\Psi _{s}(\overline{n})|^{2} (|n_1||n_2||n_3|)^{-2s-1} \\&\lesssim N_{1}^{4s+2}\cdot N_{1}^{-6s-3}\cdot N_{1}^{2} \sim N_{1}^{-2s+1}, \end{aligned}$$

which is summable as $s>\frac{1}{2}$.

This completes the estimation of (4.4) and thus also the estimates for the main part $\mathcal {Q}^{(1)}_{s,N}$.

### Contribution from $\mathcal {Q}^{(2)}_{s,N}$

We now handle the easier contribution in (4.1) coming from $\mathcal {Q}^{(2)}_{s,N}$. We further expand this operator as follows:

$$\begin{aligned} \mathcal {Q}^{(2)}_{s,N}(u)&= \langle |D_{x}|^{2s}u_{N} , \mathcal {H}(u_N^2) \rangle - \langle |D_x|^{2s}u_{N}, \mathcal {H}(1+|D_x|)^{-1} (u_N^2) \rangle \\&=: \mathcal {Q}^{(2,A)}_{s,N}(u) +\mathcal {Q}^{(2,B)}_{s,N}(u). \end{aligned}$$

Whilst the first term $\mathcal {Q}^{(2,A)}_{s,N}$ is not critical in the sense that $\mathcal {Q}^{(1)}_{s,N}$ is, there are still $\text {high-high-low}$ interactions which require *s*-derivatives to be absorbed by the high-frequency factors, which is too much for the *Y* factors to handle in a naive way. Thus, we still require some decompositions depending on the factors *Y* or *V*. As a warm up to this, we dispense with the term $\mathcal {Q}^{(2,B)}_{s,N}$.

#### Lemma 4.1

For any $\frac{1}{2}< s< 1$, it holds that

4.13 $$\begin{aligned} |\mathcal {Q}_{s,N}^{(2,B)}(u)| \lesssim \Vert u_{N}\Vert _{H^{1/2}}^{3}, \end{aligned}$$

whilst when s=1,

4.14 $$\begin{aligned} |\mathcal {Q}_{1,N}^{(2,B)}(u)| \lesssim \Vert u_{N}\Vert _{H^{1/2}}^{2} \Vert u_N\Vert _{L^{\infty }}. \end{aligned}$$

#### Proof

By rewriting and using Cauchy–Schwarz and the fractional Leibniz rule,

$$\begin{aligned} |\mathcal {Q}_{s,N}^{(2,B)}(u)|&= |\langle |D_x|^{\frac{1}{2}} u_N , \mathcal {H}|D_x|^{2s-\frac{1}{2}}(1+|D_x|)^{-1}(u_N^2) \rangle |\lesssim \Vert u_N\Vert _{H^{\frac{1}{2}}}\Vert u_{N}^{2}\Vert _{H^{2s-\frac{3}{2}}}. \end{aligned}$$

If $s\le \frac{3}{4}$, then $2s-\frac{3}{2}\le 0$ and hence (4.13) follows by Sobolev embedding $H^{\frac{1}{2}}(\mathbb {T})\hookrightarrow L^{4}(\mathbb {T})$. If $\frac{3}{4}<s<1$, then by the fractional Leibniz rule and Sobolev embedding, we have

$$\begin{aligned} \Vert u_N^2 \Vert _{H^{2s-\frac{3}{2}}} \lesssim \Vert |D_x|^{2s-\frac{3}{2}} u_N\Vert _{L^{\frac{2}{4s-3}}} \Vert u_N\Vert _{L^\frac{1}{2(1-s)}}&\lesssim \Vert u_N\Vert _{H^{\frac{1}{2}}}^2, \end{aligned}$$

which completes the proof of (4.13).

As for (4.14), we have by Cauchy–Schwarz and the fractional Leibniz rule,

$$\begin{aligned} |\mathcal {Q}_{1,N}^{(2,B)}(u)| = | \langle |D_x|^{\frac{1}{2}}u_N , \mathcal {H}|D_x|^{\frac{1}{2}}(u_N^2) \rangle | \le \Vert u_N\Vert _{H^{\frac{1}{2}}} \Vert u_N^2 \Vert _{H^{\frac{1}{2}}}^{2} \lesssim \Vert u_{N}\Vert _{H^{\frac{1}{2}}} \Vert u_N\Vert _{L^{\infty }}, \end{aligned}$$

establishing (4.14). □

Thus, it follows from Lemma 4.1 that when $\frac{1}{2}<s<1$, for $Y_N +V_N \in B_{R}$,

4.15 $$\begin{aligned} |\mathcal {Q}^{(2,B)}_{s,N}(Y_N+V_N)| \lesssim R^{\frac{3}{2}}, \end{aligned}$$

uniformly in $N\in \mathbb {N}$. When s=1, we have instead by Young’s inequality and Sobolev embedding

$$\begin{aligned} \lambda |\mathcal {Q}^{(2,B)}_{s,N}(Y_N+V_N)|&\lesssim \lambda R (\Vert Y_N\Vert _{L^{\infty }} +\Vert V_N\Vert _{L^{\infty }}) \\&\le C \lambda R\Vert Y_N\Vert _{L^{\infty }}+\lambda ^2 R^{2} C_{L} +\tfrac{1}{4L} \Vert V_{N}\Vert _{H^{s+1/2}}^2, \end{aligned}$$

where the implicit constants *C* and $C_{L}$ do not depend on $N\in \mathbb {N}$.

We move onto controlling $\mathcal {Q}^{(2,A)}_{s,N}$. We decompose each of the input functions:

4.16 $$\begin{aligned} \mathcal {Q}^{(2,A)}_{s,N}(u)&= 2 \sum _{ \begin{array}{c} N_1, N_2, N_3 \in 2^{\mathbb {N}_0} \\ N_{2}\ge N_3 \end{array}} \mathcal {Q}^{(2,A)}_{s,N}( \textbf{P}_{N_1}u, \textbf{P}_{N_2}u, \textbf{P}_{N_3}u) \nonumber \\&= 2 \sum _{ \begin{array}{c} N_1, N_2, N_3 \in 2^{\mathbb {N}_0} \\ N_{2} \sim N_3 \gtrsim N_1 \end{array}}\mathcal {Q}^{(2,A)}_{s,N}( \textbf{P}_{N_1}u, \textbf{P}_{N_2}u, \textbf{P}_{N_3}u) \end{aligned}$$

4.17 $$\begin{aligned}&+2\sum _{ \begin{array}{c} N_1, N_2, N_3 \in 2^{\mathbb {N}_0} \\ N_{2} \sim N_1 \gg N_3 \end{array}}\mathcal {Q}^{(2,A)}_{s,N}( \textbf{P}_{N_1}u, \textbf{P}_{N_2}u, \textbf{P}_{N_3}u) . \end{aligned}$$

Consider first the term (4.16), which we split into two cases. First, we assume that at least one of the highest frequency inputs is a *V*; say the $n_2$-frequency. With $u:=Y_{N}+V_N\in B_{R}$, Bernstein and Cauchy–Schwarz inequalities imply

$$\begin{aligned} |\mathcal {Q}^{(2,A)}_{s,N}( \textbf{P}_{N_1}u, \textbf{P}_{N_2}u, V_{N_3})|&\le \Vert |D_{x}|^{2s}\mathcal {H}\textbf{P}_{N_1}u\Vert _{L^{\infty }} \Vert V_{N_2} \cdot \textbf{P}_{N_3}u\Vert _{L^1} \\&\lesssim N_{1}^{2s} \big (N_{1}^{\frac{1}{2}}\Vert \textbf{P}_{N_1}u\Vert _{L^2}\big ) \Vert V_{N_2}\Vert _{L^2}\Vert \textbf{P}_{N_3}u\Vert _{L^{2}} \\&\lesssim N_{1}^{2s}N_{2}^{-s-\frac{1}{2}} N_{3}^{-\frac{1}{2}} \big (N_{1}^{\frac{1}{2}}\Vert \textbf{P}_{N_1}u\Vert _{L^2}\big ) \Vert V_{N_2}\Vert _{H^{s+\frac{1}{2}}}\Vert \textbf{P}_{N_3}u\Vert _{H^{\frac{1}{2}}}. \end{aligned}$$

Note that in the second inequality we used that $\mathcal {H}$ commutes with $\textbf{P}_{N_1}$ and is a bounded operator on $L^2$. Then, by Young’s inequality and that s≤1,

$$\begin{aligned}&\sum _{ \begin{array}{c} N_1, N_2, N_3 \in 2^{\mathbb {N}_0} \\ N_{2} \sim N_3 \gtrsim N_1 \end{array}} \lambda |\mathcal {Q}^{(2,A)}_{s,N}( \textbf{P}_{N_1}u, \textbf{P}_{N_2}u, V_{N_3})| \\&\lesssim \sum _{ \begin{array}{c} N_1, N_2, N_3 \in 2^{\mathbb {N}_0} \\ N_{2} \sim N_3 \gtrsim N_1 \end{array}} \lambda \frac{N_{1}^{s}}{N_2} \big (N_{1}^{\frac{1}{2}}\Vert \textbf{P}_{N_1}u\Vert _{L^2}\big ) \Vert V_{N_2}\Vert _{H^{s+\frac{1}{2}}}\Vert \textbf{P}_{N_3}u\Vert _{H^{\frac{1}{2}}} \\&\lesssim \sum _{N_2 \sim N_3} \Vert V_{N_2}\Vert _{H^{s+\frac{1}{2}}}\Vert \textbf{P}_{N_3}u\Vert _{H^{\frac{1}{2}}} \sum _{N_1 \le N_2} \frac{N_{1}^{s}}{N_2}\big (N_{1}^{\frac{1}{2}}\Vert \textbf{P}_{N_1}u\Vert _{L^2}\big ) \\&\lesssim \lambda R \Vert V\Vert _{H^{s+\frac{1}{2}}}\\&\le C L \lambda ^2 R + \tfrac{1}{4L} \Vert V\Vert _{H^{s+\frac{1}{2}}}^{2}, \end{aligned}$$

which is acceptable. Now, consider the case when both high-frequency inputs are Gaussians *Y*. By Cauchy–Schwarz, we have

$$\begin{aligned} |\mathcal {Q}^{(2,A)}_{s,N}( \textbf{P}_{N_1}u, Y_{N_2}, Y_{N_3})|&\lesssim \Vert |D_{x}|^{2s}\textbf{P}_{N_1}u\Vert _{L^2} \Vert \widetilde{\textbf{P}}_{N_1}\textbf{P}_{\ne 0} ( Y_{N_2} Y_{N_3})\Vert _{L^2} \\&\lesssim N_{1}^{2s-\frac{1}{2}}R^{\frac{1}{2}} \Vert \widetilde{\textbf{P}}_{N_1}\textbf{P}_{\ne 0} ( Y_{N_2} Y_{N_3})\Vert _{L^2}. \end{aligned}$$

Now, taking an expectation, we find

$$\begin{aligned}&\sum _{ \begin{array}{c} N_1, N_2, N_3 \in 2^{\mathbb {N}_0} \\ N_{2} \sim N_3 \gtrsim N_1 \end{array}} \mathbb {E}[ |\mathcal {Q}^{(2,A)}_{s,N}( \textbf{P}_{N_1}u, Y_{N_2}, Y_{N_3})| \textbf{1}_{B_{R}}(u)] \\&\lesssim R^{\frac{1}{2}} \sum _{ \begin{array}{c} N_1, N_2, N_3 \in 2^{\mathbb {N}_0} \\ N_{2} \sim N_3 \gtrsim N_1 \end{array}} N_{1}^{2s-\frac{1}{2}} \mathbb {E}[ \Vert \widetilde{\textbf{P}}_{N_1}\textbf{P}_{\ne 0} ( Y_{N_2} Y_{N_3})\Vert _{L^2}] \\&\lesssim R^{\frac{1}{2}} \sum _{ \begin{array}{c} N_1, N_2, N_3 \\ N_{2} \sim N_3 \gtrsim N_1 \end{array}} N_{1}^{2s-\frac{1}{2}} \bigg ( \sum _{\begin{array}{c} n_{123}=0 \\ |n_j|\sim N_j, j=1,2,3 \end{array}} \frac{1}{(|n_2||n_3|)^{2s+1}} \bigg )^{\frac{1}{2}}\\&\lesssim R^{\frac{1}{2}} \sum _{ \begin{array}{c} N_1, N_2, N_3 \\ N_{2} \sim N_3 \gtrsim N_1 \end{array}} N_{1}^{2s}N_{2}^{-2s-\frac{1}{2}} \lesssim R^{\frac{1}{2}}, \end{aligned}$$

uniformly in $N\in \mathbb {N}$. This completes the estimation of (4.16).

It remains to consider the contribution from the term (4.17). We split into three cases depending on the number of *V* terms appearing in the two highest frequencies (which are $N_1$ and $N_2$). First, assume that there are no *V* terms. We then claim that

4.18 $$\begin{aligned} \mathbb {E}[ \Vert \widetilde{\textbf{P}}_{N_3}\big [ \textbf{P}_{N_2}Y \cdot \mathcal {H}|D_x|^{2s}\textbf{P}_{N_1}Y \big ]\Vert _{L^2}] \lesssim N_{3}^{\frac{1}{2}}N_{1}^{-\frac{1}{2}} \quad \text {under} \quad N_1\sim N_2\gg N_3, \end{aligned}$$

uniformly in $N\in \mathbb {N}$. Indeed, by Cauchy–Schwarz, we have

$$\begin{aligned} [\text {LHS} (4.18)]^{2} \lesssim \sum _{ \begin{array}{c} n_{123}=0 \\ |n_j|\sim N_j, j=1,2,3 \end{array}} \frac{|n_1|^{4s}}{|n_1|^{2s+1}|n_2|^{2s+1}} \lesssim N_3 N_{1}^{-1}. \end{aligned}$$

Now, by Cauchy–Schwarz,

$$\begin{aligned} |\mathcal {Q}^{(2,A)}_{s,N}( \textbf{P}_{N_1}u, \textbf{P}_{N_2}u, \textbf{P}_{N_3}u) |&= | \langle \textbf{P}_{N_2}Y \cdot \mathcal {H}|D_x|^{2s}\textbf{P}_{N_1}Y, \textbf{P}_{N_3}u \rangle | \\&\lesssim N_{3}^{-\frac{1}{2}} R^{\frac{1}{2}} \Vert \widetilde{\textbf{P}}_{N_3}\big [ \textbf{P}_{N_2} Y \cdot \mathcal {H}|D_x|^{2s} \textbf{P}_{N_1}Y \big ]\Vert _{L^2} \end{aligned}$$

Then, by (4.18),

$$\begin{aligned}&\sum _{ \begin{array}{c} N_1, N_2, N_3 \in 2^{\mathbb {N}_0} \\ N_{2} \sim N_1 \gg N_3 \end{array}} 2\lambda \mathbb {E}\big [ |\mathcal {Q}^{(2,A)}_{s,N}( \textbf{P}_{N_1}Y, \textbf{P}_{N_2}Y, \textbf{P}_{N_3}(Y+V)) | \textbf{1}_{B_R}(Y+V)\big ] \\&\lesssim _{\lambda ,R} \sum _{ \begin{array}{c} N_1, N_2, N_3 \in 2^{\mathbb {N}_0} \\ N_{2} \sim N_1 \gg N_3 \end{array}} N_{1}^{-\frac{1}{2}} \lesssim _{\lambda , R} 1. \end{aligned}$$

Now, consider the case when there is exactly one *V* and one *Y*, say $Y_{N_1}$ and $V_{N_2}$. The remaining case is dealt with in the same way since $N_1 \sim N_2$. Then, by Bernstein and Young inequalities, we have

$$\begin{aligned} \lambda |\mathcal {Q}^{(2,A)}_{s,N}( Y_{N_1}, V_{N_2}, \textbf{P}_{N_3}u) |&\lesssim \lambda \Vert V_{N_2}\cdot \mathcal {H}|D_x|^{2s}Y_{N_1}\Vert _{L^1} (N_{3}^{\frac{1}{2}}\Vert \textbf{P}_{N_3}u\Vert _{L^2}) \\&\lesssim \lambda N_{2}^{-s-\frac{1}{2}}N_{1}^{2s}R \Vert Y_{N_1}\Vert _{L^2} \Vert V_{N_2}\Vert _{H^{s+\frac{1}{2}}} \\&\le C_{\varepsilon } \lambda ^2 N_{1}^{2s-1+\delta } R^{2} \Vert Y_{N_1}\Vert _{L^2}^{2} + \varepsilon N_{1}^{-\delta }\Vert V\Vert _{H^{s+\frac{1}{2}}}^{2}, \end{aligned}$$

where 0<δ<1 and $\varepsilon =\varepsilon (s,\delta )>0$ such that

$$\begin{aligned} 2L \varepsilon \sum _{ \begin{array}{c} N_1, N_2, N_3 \in 2^{\mathbb {N}_0} \\ N_{2} \sim N_1 \gg N_3 \end{array}}N_{1}^{-\delta } \le \frac{1}{4L}. \end{aligned}$$

For the first term, we then get

$$\begin{aligned} N_{1}^{2s-1+\delta } \mathbb {E}[ \Vert Y_{N_1}\Vert _{L^2}^{2} ] \lesssim N_{1}^{-1+\delta } \end{aligned}$$

which is summable.

Finally, we have the case where both high-frequency factors are *V* terms. Similar to the previous case, we have

$$\begin{aligned} |\mathcal {Q}^{(2,A)}_{s,N}( V_{N_1}, V_{N_2}, \textbf{P}_{N_3}u) |&\lesssim \Vert V_{N_1}\cdot \mathcal {H}|D_x|^{2s}V_{N_2}\Vert _{L^1} (N_{3}^{\frac{1}{2}}\Vert \textbf{P}_{N_3}u\Vert _{L^2}) \\&\lesssim RN_1^{s-\frac{1}{2}} \Vert \textbf{P}_{N_1}V\Vert _{L^2} \Vert V\Vert _{H^{s+\frac{1}{2}}}. \end{aligned}$$

If s<1, then $s-\frac{1}{2} <\frac{1}{2}$ and we have

$$\begin{aligned} |\mathcal {Q}^{(2,A)}_{s,N}( V_{N_1}, V_{N_2}, \textbf{P}_{N_3}u) | \lesssim N_{1}^{-s+1} R^{2} \Vert V\Vert _{H^{s+\frac{1}{2}}}. \end{aligned}$$

By repeating the Cauchy–Schwarz argument from the previous case, we control the contribution of this term. If s=1, by (4.2) we have

$$\begin{aligned} \lambda |\mathcal {Q}^{(2,A)}_{s,N}( V_{N_1}, V_{N_2}, \textbf{P}_{N_3}u) |&\lesssim \lambda R^{\frac{1}{2}} N_1^{2} \Vert V_{N_1}\Vert _{L^2} \Vert V_{N_2}\Vert _{L^2} \\&\lesssim \lambda R^{\frac{1}{2}}N_{1}^{-\frac{1}{4}} \Vert V\Vert _{H^{\frac{1}{2}}}^{\frac{3}{4}} \Vert V\Vert _{H^{\frac{3}{2}}}^{\frac{5}{4}}\\&\lesssim \lambda R^{\frac{1}{2}} N_1^{-\frac{1}{4}}(R+\Vert Y\Vert _{H^{\frac{1}{2}}})^{\frac{3}{8}} \Vert V\Vert _{H^{\frac{3}{2}}}^{\frac{5}{4}} \\&\le C_{\varepsilon }\lambda ^{\frac{8}{5}} R^{\frac{4}{5}} N_{1}^{-\frac{1}{5}} (R+\Vert Y\Vert _{H^{\frac{1}{2}}})^{\frac{3}{5}} + \varepsilon N_{1}^{-\frac{1}{3}} \Vert V\Vert _{H^{\frac{3}{2}}}^{2}. \end{aligned}$$

for any ε>0. Thus, the contribution from this term is controlled after performing the dyadic summations and choosing ε>0 sufficiently small depending on *L*.

This completes the estimation of $\mathcal {Q}^{(2,A)}_{s,N}$ and thus the proof of Lemma 3.3.

### Proof of Proposition 1.3

We revisit some of the computations we made in the previous sections to show the divergent lower bounds. Let $0<s\le \frac{1}{2}$, *N* fixed to be chosen large enough later, and suppose t≠0. By the decomposition (3.5) and Cauchy’s inequality, we have

4.19 $$\begin{aligned} \text {QI}_{s,N} \ge (1-\varepsilon ) \mathbb {E}\bigg [ \bigg | \int _{0}^{t} Q^{(1)}_{s,N}( S(-t') u_0^{\omega })dt'\bigg |^{2}\bigg ] -C_{\varepsilon } \mathbb {E}\bigg [ \bigg | \int _{0}^{t} Q^{(2)}_{s,N}( S(-t') u_0^{\omega })dt'\bigg |^{2}\bigg ], \end{aligned}$$

for any ε>0. Now,

$$\begin{aligned} \int _{0}^{t} Q^{(2)}_{s,N}( S(-t') u_0^{\omega })dt'= \sum _{ \begin{array}{c} n_{123}=0 \\ 0<|n_j|\le N, \, j=1,2,3 \end{array}} \frac{|n_1|^{2s}n_1}{1+|n_1|} \frac{e^{it\Phi (\overline{n})}-1}{\Phi (\overline{n})} \prod _{j=1}^{3} \frac{g_{n_j}}{|n_j|^{s+1/2}}, \end{aligned}$$

where

4.20 $$\begin{aligned} \Phi (\overline{n}):= \Phi (n_1,n_2,n_3) : = \frac{n_1}{1+|n_1|} +\frac{n_2}{1+|n_2|} +\frac{n_3}{1+|n_3|}. \end{aligned}$$

By the Isserlis’ theorem, we have

4.21 $$\begin{aligned} \begin{aligned} \mathbb {E}\bigg [ \bigg | \int _{0}^{t} Q^{(2)}_{s,N}( S(-t') u_0^{\omega })dt'\bigg |^{2}\bigg ]&\lesssim t^{2} \bigg (\sum _{n_{123}=0} \frac{|n_1|^{4s}+|n_1|^{2s} |n_{2}|^{2s} }{(|n_1||n_2||n_3|)^{2s+1}} \bigg ) \lesssim t^2. \end{aligned} \end{aligned}$$

Next, we also have

4.22 $$\begin{aligned} \mathbb {E}\bigg [ \bigg | \int _{0}^{t} Q^{(1)}_{s,N}( S(-t') u_0^{\omega })dt'\bigg |^{2}\bigg ]&\sim \sum _{ \begin{array}{c} n_{123}=0 \\ 0<|n_j|\le N, \, j=1,2,3 \end{array}} \frac{| \Psi _{s}(\overline{n})|^{2}}{(|n_1||n_2||n_3|)^{2s+1}} \bigg | \frac{e^{it\Phi (\overline{n})}-1}{\Phi (\overline{n})} \bigg |^{2} \nonumber \\&\gtrsim t^2 \sum _{ \begin{array}{c} n_{123}=0 \\ 0<|n_j|\le N, \, j=1,2,3 \end{array}} \frac{| \Psi _{s}(\overline{n})|^{2}}{(|n_1||n_2||n_3|)^{2s+1}}, \end{aligned}$$

where we used that $|\Phi (\overline{n})| \lesssim 1$ for all $(n_1,n_2,n_3)$ and the assumption |t|≲1.

We now split into two cases. First, suppose that $0<s<\frac{1}{2}$. Let $1\le A \ll N$ to be chosen later. Consider the subset of frequency interactions where $n_1\in \frac{1}{2} N + [\frac{3}{4} A, A]$, $n_{2}\in -\frac{1}{2} N + [0,\frac{1}{4} A]$. Then, $n_3\in [-\frac{5}{4} A, -\frac{3}{4}A]$ and by the mean value theorem,

4.23 $$\begin{aligned} |\Psi _{s}(\overline{n})| \gtrsim N^{2s} A -A^{2s+1} \gtrsim A N^{2s}. \end{aligned}$$

Thus,

$$\begin{aligned} (4.22)&\gtrsim t^{2} A^{1-2s}N^{-2} \big | \{ (n_1,n_2,n_3) \in \mathbb {Z}_{*} \, : \, n_{123}=0, \,\, n_1\in \tfrac{1}{2} N + [\tfrac{3}{4} A, A] , \\&\quad n_{2}\in -\tfrac{1}{2} N + [0,\tfrac{1}{4} A], \, n_3 \in [-\tfrac{5}{4} A, -\tfrac{3}{4}A]\} \big | \\&\sim t^{2} A^{3-2s}N^{-2}. \end{aligned}$$

Choose A=δN for any $0<\delta \ll \frac{1}{2}$ so that (4.23) holds. Then, we choose *N* large so that $N\gg \widetilde{C} C_{\varepsilon }$, where $\widetilde{C}$ is the implicit constant in (4.21) at which point by (4.19) we obtain (1.15).

Now, assume that $s=\frac{1}{2}$. On the subset

$$\begin{aligned} \Gamma&:= \{ (n_1,n_2,n_3)\in \mathbb {Z}_{*} \, : \, n_{123}=0,\,\, |n_j| \le N, \\ j&=1,2,3, \,\, n_2,n_3<0<n_1, |n_1|\sim |n_2|\gg |n_3|\}, \end{aligned}$$

we have

$$\begin{aligned} \Psi _{\frac{1}{2}}(\overline{n})=n_1^{2}-n_2^2 -n_3^2= -n_3(n_1-n_2+n_3)= |n_3|(|n_1|+|n_2|-|n_3|). \end{aligned}$$

Thus,

$$\begin{aligned} (4.22) \gtrsim t^{2} \sum _{\Gamma } \frac{| \Psi _{\frac{1}{2}}(\overline{n})|^{2}}{(n_1 n_2 n_3)^{2}} \gtrsim t^{2} \sum _{\Gamma } \frac{1}{n_2^{2}}-t^{2} \sum _{\Gamma } \frac{1}{n_1^2 n_2^2} \sim t^{2} (\log N)^2. \end{aligned}$$

This completes the $s=\frac{1}{2}$ case of (1.15) and thus the proof of proof of Proposition 1.3.

## Deterministic growth bound

In this section, we prove Theorem 1.1. We first obtain a bound for the time derivative of $\Vert \textbf{P}_{N}u\Vert _{H^\sigma }^2$, for *u* a smooth solution to (1.1). We have

$$\begin{aligned} \frac{d}{dt} \Big ( \tfrac{1}{2}\Vert \textbf{P}_{N}u\Vert _{H^\sigma }^2\Big )&= \langle |D_x|^{2\sigma -1}\textbf{P}_{N}u , \partial _x\textbf{P}_{N}(u^2) \rangle - \langle |D_x|^{2\sigma -1}\textbf{P}_{N}u , (1+|D_x|)^{-1}\partial _x\textbf{P}_{N}(u^2) \rangle \\&=: A_1 +A_2. \end{aligned}$$

For the easier term $A_2$, we use Cauchy–Schwarz and that

5.1 $$\begin{aligned} \textbf{P}_{N}(u^2)= \textbf{P}_{N}(u_{\gtrsim N}^{2} + 2 u_{\ll N}u) \end{aligned}$$

to obtain

5.2 $$\begin{aligned} |A_2|&\lesssim \Vert |D_x|^{2\sigma -1} \textbf{P}_{N}u\Vert _{L^2} \Vert \textbf{P}_{N}(u^2)\Vert _{L^2} \nonumber \\&\lesssim N^{2\sigma -\frac{3}{2}} \Vert \textbf{P}_{N}u\Vert _{H^{\frac{1}{2}}} \Vert u_{\gtrsim N}\Vert _{L^4}^2 +N^{\sigma -1}\Vert \textbf{P}_{N}u\Vert _{H^\sigma } \Vert u_{\ll N}\Vert _{L^{\infty }}\Vert \widetilde{\textbf{P}}_{N}u\Vert _{L^2}\nonumber \\&\lesssim N^{2\sigma -\frac{3}{2}} \Vert \textbf{P}_{N}u\Vert _{H^{\frac{1}{2}}} (N^{-\sigma +\frac{1}{4}} \Vert u\Vert _{H^\sigma })^2 + N^{-1+} \Vert u\Vert _{H^{\frac{1}{2}}} \Vert \textbf{P}_{N}u\Vert _{H^\sigma } \Vert \widetilde{\textbf{P}}_{N}u\Vert _{H^\sigma } \nonumber \\&\lesssim N^{-1+} \Vert u\Vert _{H^{\frac{1}{2}}} \Vert u\Vert _{H^\sigma }^2. \end{aligned}$$

Now, we handle $A_1$. We use (5.1) to find

$$\begin{aligned} A_1&= \langle |D_x|^{2\sigma -1}\textbf{P}_{N}u, \partial _x\textbf{P}_{N}(u_{\gtrsim N}^2) \rangle +2 \langle |D_x|^{2\sigma -1}\textbf{P}_{N}u, \partial _x\textbf{P}_{N}( u_{\ll N} u) \rangle \\&=\langle |D_x|^{2\sigma -1}\textbf{P}_{N}u, \partial _x\textbf{P}_{N}(u_{\gtrsim N}^2) \rangle + 2 \langle |D_x|^{2\sigma -1}\textbf{P}_{N}u, \textbf{P}_{N}( (\partial _xu_{\ll N}) u) \rangle \\&\quad + 2 \langle |D_x|^{\sigma -\frac{1}{2}}\textbf{P}_{N}u, u_{\ll N} |D_x|^{\sigma -\frac{1}{2}}\partial _x\textbf{P}_{N}u \rangle + 2 \langle |D_x|^{\sigma -\frac{1}{2}}\textbf{P}_{N}u, [ |D_x|^{\sigma -\frac{1}{2}}\textbf{P}_{N}, u_{\ll N}] \partial _xu \rangle \\&=: A_{11}+A_{12}+A_{13}+A_{14}, \end{aligned}$$

where $[ |D_x|^{\sigma -\frac{1}{2}}\textbf{P}_{N}, u_{\ll N}]f= |D_x|^{\sigma -\frac{1}{2}}\textbf{P}_{N}( u_{\ll N}f) - u_{\ll N}|D_x|^{\sigma -\frac{1}{2}}\textbf{P}_{N} f$ denotes the commutator.

For $A_{11}$, by Sobolev embedding, we have

5.3 $$\begin{aligned} |A_{11}|&\lesssim N^{2\sigma -\frac{1}{2}}\Vert \textbf{P}_{N}u\Vert _{H^{\frac{1}{2}}} \Vert u_{\gtrsim N}\Vert _{H^{\frac{1}{4}}}^2 \lesssim \sum _{M\gtrsim N} \bigg ( \frac{N}{M}\bigg )^{2\sigma -\frac{1}{2}}\Vert \textbf{P}_{M}u\Vert _{H^\sigma }^2 \Vert \textbf{P}_{N}u\Vert _{H^{\frac{1}{2}}}. \end{aligned}$$

To handle, the remaining terms, we note the following estimate:

5.4 $$\begin{aligned} N^{-1} \Vert \partial _xu_{\ll N}\Vert _{L^{\infty }} \lesssim \sum _{M\ll N}N^{-1}M \Vert \textbf{P}_{M}u\Vert _{H^{\frac{1}{2}}} \lesssim \bigg ( \sum _{M\ll N} N^{-2}M^2 \bigg )^{\frac{1}{2}} \Vert u\Vert _{H^{\frac{1}{2}}} \lesssim \Vert u\Vert _{H^{\frac{1}{2}}}. \end{aligned}$$

Then, by Bernstein’s inequality and (5.4), we have

5.5 $$\begin{aligned} |A_{12}|&\lesssim \Vert |D_x|^{2\sigma -1}\textbf{P}_{N}u\Vert _{L^2} \Vert \partial _xu_{\ll N}\Vert _{L^{\infty }} \Vert \widetilde{\textbf{P}}_{N}u\Vert _{L^2} \nonumber \\&\lesssim N^{-1}\Vert \partial _xu_{\ll N}\Vert _{L^{\infty }} \Vert \textbf{P}_{N}u\Vert _{H^\sigma } \Vert \widetilde{\textbf{P}}_{N}u\Vert _{H^\sigma } \nonumber \\&\lesssim \Vert u\Vert _{H^{\frac{1}{2}}} \Vert \textbf{P}_{N}u\Vert _{H^\sigma } \Vert \widetilde{\textbf{P}}_{N}u\Vert _{H^\sigma }. \end{aligned}$$

For $A_{13}$, integration by parts and (5.4) imply

5.6 $$\begin{aligned} |A_{13}|&=2| \langle (| D_x|^{\sigma -\frac{1}{2}}\textbf{P}_{N}u)^2 , \partial _xu_{\ll N} \rangle |\lesssim N^{-1}\Vert \partial _xu_{\ll N}\Vert _{L^{\infty }} \Vert \textbf{P}_{N}u\Vert _{H^\sigma }^2 \nonumber \\&\lesssim \Vert u\Vert _{H^{\frac{1}{2}}} \Vert \textbf{P}_{N}u\Vert _{H^\sigma }. \end{aligned}$$

Lastly, we consider $A_{14}$. Notice that by frequency considerations, we have

$$\begin{aligned} A_{14}=2 \langle |D_x|^{\sigma -\frac{1}{2}}\textbf{P}_{N}u, [ |D_x|^{\sigma -\frac{1}{2}}\textbf{P}_{N}, u_{\ll N}] \partial _x\widetilde{\textbf{P}}_{N} u \rangle \end{aligned}$$

where the last factor of *u* was replaced by $\widetilde{\textbf{P}}_{N}u$. Then, by a dyadic decomposition, Cauchy–Schwarz and Minkowski inequality, Plancherel’s theorem, and Young’s inequality, we have

5.7 $$\begin{aligned} |A_{14}|&\lesssim \Vert \textbf{P}_{N}u\Vert _{H^{\sigma -\frac{1}{2}}} \sum _{M\ll N} \Big \Vert [|D_x|^{\sigma -\frac{1}{2}}\textbf{P}_{N}, \textbf{P}_{M}u] \partial _x\widetilde{\textbf{P}}_{N} u\Big \Vert _{L^2} \nonumber \\&\lesssim N^{-\frac{1}{2}}\Vert \textbf{P}_{N}u\Vert _{H^\sigma } \sum _{M\ll N} \nonumber \\&\quad \bigg \Vert \sum _{n=n_1+n_2} in_2 \big ( |n|^{\sigma -\frac{1}{2}}\phi (\tfrac{n}{N}) - |n_2|^{\sigma -\frac{1}{2}}\phi (\tfrac{n_2}{N}) \big ) \widehat{\textbf{P}_{M}u}(n_1) \widehat{ \widetilde{\textbf{P}}_{N}u}(n_2)\bigg \Vert _{\ell ^{2}_{n}}\nonumber \\&\lesssim N^{-\frac{1}{2}}\Vert \textbf{P}_{N}u\Vert _{H^\sigma } \sum _{M\ll N} N^{\sigma -\frac{1}{2}} M\bigg \Vert \sum _{n=n_1+n_2} | \widehat{\textbf{P}_{M}u}(n_1)| | \widehat{ \widetilde{\textbf{P}}_{N}u}(n_2) |\bigg \Vert _{\ell ^{2}_{n}} \nonumber \\&\lesssim N^{-\frac{1}{2}}\Vert \textbf{P}_{N}u\Vert _{H^\sigma } \sum _{M\ll N} N^{\sigma -\frac{1}{2}} M \Vert \widehat{\textbf{P}_{M}u}\Vert _{\ell ^1_n} \Vert \widehat{ \widetilde{\textbf{P}}_{N}u}\Vert _{\ell ^2_n}\nonumber \\&\lesssim \Vert \textbf{P}_{N}u\Vert _{H^\sigma } \Vert \widetilde{\textbf{P}}_{N}u\Vert _{H^\sigma } \sum _{M\ll N} N^{-1}M\Vert \textbf{P}_{M}u\Vert _{H^{\frac{1}{2}}}\nonumber \\&\lesssim \Vert u\Vert _{H^{\frac{1}{2}}}\Vert \textbf{P}_{N}u\Vert _{H^\sigma } \Vert \widetilde{\textbf{P}}_{N}u\Vert _{H^\sigma }, \end{aligned}$$

where in the third inequality we used the mean value theorem to obtain

$$\begin{aligned} ||n|^{\sigma -\frac{1}{2}}\phi (\tfrac{n}{N}) - |n_2|^{\sigma -\frac{1}{2}}\phi (\tfrac{n_2}{N}) | \lesssim N^{\sigma -\frac{3}{2}}M. \end{aligned}$$

Combining (5.2), (5.3), (5.5), (5.6), (5.7), the fundamental theorem of calculus, and the conservation of $\Vert u(t)\Vert _{H^{\frac{1}{2}}}$, we get

5.8 $$\begin{aligned} \begin{aligned} \Vert \textbf{P}_{N}u(t)\Vert _{H^{\sigma }}^{2} \lesssim \Vert \textbf{P}_{N}&u(0)\Vert _{H^\sigma }^2 + \int _{0}^{t} \sum _{M\gtrsim N} \bigg ( \frac{N}{M}\bigg )^{2\sigma -\frac{1}{2}}\Vert \textbf{P}_{M}u(t')\Vert _{H^\sigma }^2 \Vert \textbf{P}_{N}u(t')\Vert _{H^{\frac{1}{2}}} dt'\\&+\int _{0}^{t} \Vert u(0)\Vert _{H^{\frac{1}{2}}} \big [ N^{-1+}\Vert u(t')\Vert _{H^\sigma }^2 + \Vert \textbf{P}_{N}u(t')\Vert _{H^\sigma } \Vert \widetilde{\textbf{P}}_{N}u(t')\Vert _{H^\sigma }] dt' . \end{aligned} \end{aligned}$$

Summing both sides over dyadic *N* and using that, for $\sigma >\frac{1}{4}$,

$$\begin{aligned}&\sum _{N} \Vert \textbf{P}_{N}u\Vert _{H^\sigma } \Vert \widetilde{\textbf{P}}_{N}u\Vert _{H^\sigma } \lesssim \Vert u\Vert _{H^\sigma }^2 , \\&\sum _{N} \sum _{M\gtrsim N} \bigg ( \frac{N}{M}\bigg )^{2\sigma -\frac{1}{2}}\Vert \textbf{P}_{M}u\Vert _{H^\sigma }^2 \Vert \textbf{P}_{N}u(t')\Vert _{H^{\frac{1}{2}}} \\&= \sum _{M} \Vert \textbf{P}_{M}u\Vert _{H^\sigma }^2 \sum _{N\lesssim M}\bigg ( \frac{N}{M}\bigg )^{2\sigma -\frac{1}{2}} \Vert \textbf{P}_{N}u\Vert _{H^{\frac{1}{2}}} \\&\lesssim \Vert u\Vert _{H^{\sigma }}^2 \Vert u\Vert _{H^{\frac{1}{2}}}, \end{aligned}$$

we then obtain

$$\begin{aligned} \Vert u(t)\Vert _{H^{\sigma }}^2 \lesssim \Vert u(0)\Vert _{H^\sigma }^{2} +\int _{0}^{t} \Vert u(0)\Vert _{H^{\frac{1}{2}}} \Vert u(t')\Vert _{H^{\sigma }}^{2} dt', \end{aligned}$$

and hence (1.6) follows by Gronwall’s inequality. This completes the proof of Theorem 1.1.

## Data Availability

Data sharing was not applicable to this article as no datasets were generated or analysed during the current study.

## Footnotes

1 Strictly speaking, (1.7) was proved for solutions to a truncated version of (1.1) but we ignore this technicality in this discussion.

2 See (4.12).
